# hECA: The cell-centric assembly of a cell atlas

**DOI:** 10.1016/j.isci.2022.104318

**Published:** 2022-04-28

**Authors:** Sijie Chen, Yanting Luo, Haoxiang Gao, Fanhong Li, Yixin Chen, Jiaqi Li, Renke You, Minsheng Hao, Haiyang Bian, Xi Xi, Wenrui Li, Weiyu Li, Mingli Ye, Qiuchen Meng, Ziheng Zou, Chen Li, Haochen Li, Yangyuan Zhang, Yanfei Cui, Lei Wei, Fufeng Chen, Xiaowo Wang, Hairong Lv, Kui Hua, Rui Jiang, Xuegong Zhang

**Affiliations:** 1 MOE Key Lab of Bioinformatics, Bioinformatics Division of BNRIST and Department of Automation, Tsinghua University, Beijing 100084, China; 2 Fuzhou Institute of Data Technology, Changle, Fuzhou 350200, China; 3 School of Medicine, Tsinghua University, Beijing 100084, China; 4 School of Life Sciences, Center for Synthetic and Systems Biology, Tsinghua University, Beijing 100084, China

**Keywords:** Cell biology, Stem cells research, Bioinformatics

## Abstract

The accumulation of massive single-cell omics data provides growing resources for building biomolecular atlases of all cells of human organs or the whole body. The true assembly of a cell atlas should be cell-centric rather than file-centric. We developed a unified informatics framework for seamless cell-centric data assembly and built the human Ensemble Cell Atlas (hECA) from scattered data. hECA v1.0 assembled 1,093,299 labeled human cells from 116 published datasets, covering 38 organs and 11 systems. We invented three new methods of atlas applications based on the cell-centric assembly: “*in data*” cell sorting for targeted data retrieval with customizable logic expressions, “quantitative portraiture” for multi-view representations of biological entities, and customizable reference creation for generating references for automatic annotations. Case studies on agile construction of user-defined sub-atlases and “*in data*” investigation of CAR-T off-targets in multiple organs showed the great potential enabled by the cell-centric ensemble atlas.

## Highlights

• A unified informatics framework for seamless cell-centric assembly of massive single-cell data
• Built the general-purpose human Ensemble Cell Atlas (hECA) V1.0 from scattered data
• Three new methods of applications enabling “*in data*” cell experiments and portraiture
• Case studies of agile atlas reconstruction and target therapies side-effect discovery

## Graphical abstract

**Figure:**
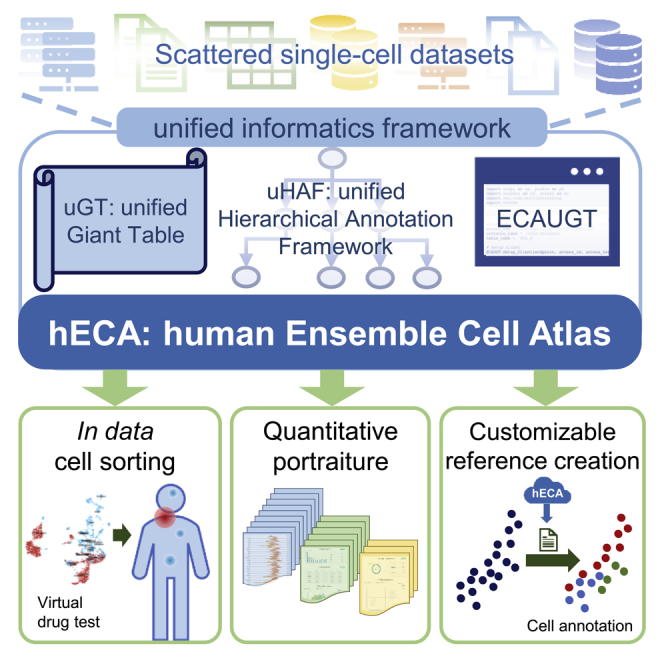

## Introduction

Cells are the basic structural and functional units of the human body. Different types of cells in different tissues and organs of the human body could be characterized by their various molecular features, especially transcriptomic features. Building molecular atlases at single-cell resolution of all cell types in the human body of health or disease can provide basic references for future biomedical studies. The HCA (Human Cell Atlas) and the HuBMAP (Human BioMolecular Atlas Program) (Regev et al., 2017; Snyder et al., 2019) are two major efforts for building such references, among several other projects aiming at similar or related goals. These big consortiums have involved labs worldwide in generating and organizing data (Arazi et al., 2019; Azizi et al., 2018; Bayraktar et al., 2020; Chevrier et al., 2017; Cillo et al., 2020; Corridoni et al., 2020; Fernandez et al., 2019; Grubman et al., 2019; Vieira Braga et al., 2019; Villani et al., 2017; Wang et al., 2020a).

The rapid development and democratization of single-cell technologies have propelled a wave of single-cell studies. Massive amounts of single-cell transcriptomic data are pouring into the public domain. Data from these studies have covered all major adult human organs (e.g., Aizarani et al., 2019; Bayraktar et al., 2020; Guo et al., 2018; Han et al., 2020; Litviňuková et al., 2020; Pellin et al., 2019), key developmental stages (e.g., Asp et al., 2019; Cao et al., 2020; Cui et al., 2019; Guo et al., 2020; Kernfeld et al., 2018; Park et al., 2020; Zhong et al., 2018), and samples from healthy donors and patients in disease (e.g., Grubman et al., 2019; Reyfman et al., 2019; Wang et al., 2020a; Zhang et al., 2020). Most single-cell studies have generated data for their specific scientific questions rather than for building atlases. But these scattered public single-cell data suggest an alternative approach of building cell atlases in a bottom-up “shot-gun” manner if data can be assembled from multiple sources. Such assembly should be cell-centric, i.e., cells from different sources should be unified into the same data repository rather than stored and indexed as a collection of files.

Assembling data of massive amounts of cells from multiple sources into an ensemble atlas have many technical and conceptual challenges (S. Chen et al., 2022b). Firstly, single-cell omics data describe the abundances and occurrences of a large variety of molecules and molecular events in many single cells. The data dimensionality and volume require very wide and long sample-by-feature tables for storage. Traditional relational databases fail to hold data of such sizes. Special infrastructure adaptable for ensemble storage and efficient retrieval of massive single-cell data is needed. Secondly, a universal indexing scheme for cells in the human body is lacking. At the macroscopic level, cells can be indexed by their anatomic and spatial arrangements, such as organs and regions. But the microscopic location of each cell is not deterministic or destined. Multiple factors or properties may be used to index the cells at different granularities for different study purposes. It is not feasible to form one fixed coordinate system to index all cells in an atlas. In addition, current annotations of cell type labels in different studies are not consistent. A standard vocabulary system for fine-grained cell identity annotation is still lacking. A unified informatics framework is needed to tackle these challenges (Börner et al., 2021; S. Chen et al., 2022b; Osumi-Sutherland et al., 2021).

We developed the human Ensemble Cell Atlas (hECA) as an instance of such a unified informatics framework. In hECA v1.0, we collected the single-cell transcriptomic data of 1,093,299 cells from 116 published datasets, covering 38 human organs and 146 cell types. hECA realized the cell-centric assembly of these data into a unified data repository with a special storage engine called uGT or unified giant table. It has the capacity to contain all possible attributes that could be used as indexes of the cells besides the transcriptomic data. The “assembly” of a cell atlas is the unified storage and organization of all the information, rather than ordering the cells with only one fixed coordinate system. Such cell-centric assembly allows for multiple ways of indexing the cells in the atlas. Along with uGT is a unified hierarchical annotation framework (uHAF) we developed for hECA. Annotating with uHAF makes cell type labels from different datasets comparable and consistent. We also developed an API named ECAUGT (pronounced “e-caught”) to retrieve cells in the atlas efficiently. With these technologies, we developed three new schemes for comprehensive application of the assembled atlas: (1) “*in data*” cell sorting for selecting cells from the virtual human body of the assembled cells using flexible combinations of logic expressions, (2) a “quantitative portraiture” system for representing the complete information of genes, cell types, and organs, and (3) “customizable reference creation” for users to customize their references for cell type annotation tasks. We conducted a series of experiments to verify and illustrate the quality and usability of the assembled data in multiple application scenarios. Especially, case examples on the agile construction of specific sub-atlases and *in data* investigation of drug off-targets throughout the whole body showed that the hECA opens many new possibilities in biomedical research using the ensemble cell atlas.

## Results

### Overview of hECA v1.0

Unlike genomes, elements in cell atlases cannot be indexed or arranged in a simple linear order or a deterministic 3D coordinate system. There are many possible ways of logical arrangements of cells at multiple granularities. The assembly of a cell atlas should convey the multifaceted nature of the data and allow users to search with customized conditions among different indexing methods.

We reasoned that the ideal cell atlas assembly should have the following properties: all cells and their multifaceted indexing coordinates should be deposited in one data system; the data system should support flexible searching using any indexing criteria, thus enabling viewing and utilizing the atlas at multiple possible angles and resolutions. The system should be “cell-centric” in the sense that cells rather than datasets or files are the basic unit of data deposit, organization, and retrieval (S. Chen et al., 2022b).

We developed such a system called human Ensemble Cell Atlas or hECA by assembling single-cell RNA-seq data collected from scattered literature. The data sources include large projects such as the Human Cell Landscape (Han et al., 2020) and Allen Brain Atlas (Sunkin et al., 2013), as well as smaller datasets in many other publications (details of current data sources are given in Table S1). We collected and processed the data with standardized quality control and normalization described in STAR Methods. The current version (hECA v1.0) contains data of 1,093,299 cells covering 38 human organs and 11 systems (integumentary, endocrine, urinary, cardiovascular, lymphatic, nervous, respiratory, digestive, muscular, reproductive, and skeletal systems). All cells were annotated with a unified framework of 146 cell type labels. The design of the system allows for the inclusion of magnitudes larger number of cells and cell types in future versions. Table 1 summarizes the current numbers of collected cells in each organ.

**Table 1:** Summary of cells collected in the organs in hECA v1.0

#	Organ	# of cells	#	Organ	# of cells
1	Adipose	1,362	20	Oesophagus	87,947
2	Adrenal gland	15,065	21	Ovary	6,927
3	Bladder	3,980	22	Pancreas	26,566
4	Blood	29,514	23	Placenta	9,926
5	Bone marrow	8,671	24	Pleura	19,695
6	Brain	214,314	25	Prostate	2,445
7	Bronchi	12,553	26	Rectum	5,718
8	Colon	22,919	27	Rib	5,907
9	Duodenum	3,743	28	Skin	6,618
10	Eye	47,275	29	Spinal cord	4,483
11	Gallbladder	14,733	30	Spleen	15,806
12	Heart	210,597	31	Stomach	22,187
13	Ileum	3,132	32	Testis	13,210
14	Intestine	41,851	33	Thymus	4,516
15	Jejunum	4,198	34	Thyroid	12,599
16	Kidney	45,368	35	Ureter	2,205
17	Liver	26,475	36	Uterine tube	6,496
18	Lung	90,521	37	Uterus	8,096
19	Muscle	26,029	38	Vessel	9,652

**Figure 1:**
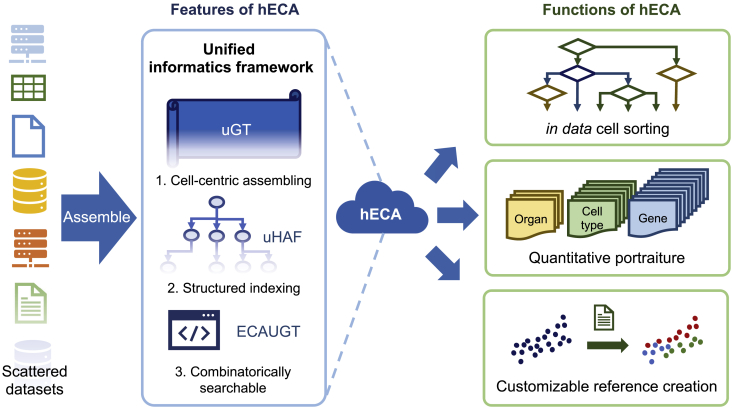
Overview of hECA. Scattered data are assembled into the ensemble cell atlas using a unified informatics framework The framework includes three key components uGT, uHAF, and ECAUGT. They made hECA the first cell-centric assembled cell atlases with structured indexing and support for combinatorial searching. Based on these components, three novel functions were built on hECA: “*in data*” cell sorting, quantitative portraiture, and customizable reference creation. See also Figures S5, S6, and S7.

The overall conceptual structure of hECA is illustrated in Figure 1. It is an instance of the ideal unified informatics framework required for cell atlas assembly (S. Chen et al., 2022b). It includes three key components: a unified giant table (uGT), a unified hierarchical annotation framework (uHAF), and an API ECAUGT for retrieving data, and three novel applications enabled by these components. uGT is a unified storage system that is technically unbounded in both rows and columns for future increases in cell numbers and feature dimensions. The basic storage of hECA is flattened to a millions-by-billions giant table (designed scale, the current instance in hECA v1.0 is 43,878 by 1,093,299). All features and metadata (any related information such as tissue origin, donor description, data source, etc., see STAR Methods for complete list) of every single cell are stored together. This unified storage strategy allows instant access to all information of every cell, enables flexible ways of retrieving, analyzing, and comparing data, and breaks the boundary of data sources while preserving the original information. uHAF is a structured knowledge graph serving as an underlying index system for hECA. This structure organizes data into a hierarchy, providing perspectives for representing relations and interactions between entities in biological knowledge while preserving space for future knowledge and data growth. We provided quantitative portraits of all existing entities in this structure and a tree-view filter of the structure for cell sorting. uHAF is designed to be compatible with other cell ontology systems and open to future upgrades. ECAUGT is a multi-functional API (application programming interface) for manipulating data in hECA (Source code: https://pypi.org/project/ECAUGT/). Based on it, we built hECA as a highly interactive system with both a graphical user interface and command-line tools. Users can access data and structured annotations with these interfaces for downstream applications. The web interface has provided useful tools for browsing, visualizing, summarizing, and analyzing pre-selected or user-selected data in hECA. Advanced users can write codes with the API for more sophisticated re-organization and deeper analyses of the data. Details of uGT, uHAF, and ECAUGT are given in STAR Methods.

Based on these technologies and the assembled data, we invented three novel ways of using cell atlases for comprehensive biomedical investigations. We developed an “*in data*” cell sorting technology that takes the assembled atlas as a virtual human body to select cells from with advanced logic conditions. We developed a “quantitative portraiture” system for representing biological entities involved in the atlas from multiple angles in a holographic manner instead of only using a few marker genes. We provided the feature of customizable reference creation for the basic application of using cell atlas data to annotate users’ in-house data. Users can define their own logic combinations to select and organize cells in hECA to form the reference for their specific queries.

### Data quality validation

hECA aims to assemble the cell-centric atlas with all accessible published data scattered in different studies, sampled from different donors by different labs, and sequenced with different technologies. These are major factors that may cause batch effects (Luecken et al., 2022), along with other known or unknown factors. The batch-effect issue is a big challenge for single-cell expression studies. There have been several published methods for batch-effect correction (Luecken et al., 2022). They were all designed for particular downstream analysis tasks with certain assumptions. The definition of batch-effect depends on specific application scenarios. A factor regarded as a batch effect to be removed in one study can be a factor of biological interest in another investigation. Existing batch-effect correction methods were not designed to serve all possible downstream applications. In hECA v1.0, we adopted a library-size-based normalization method in the data preprocessing to remove the most explained data variance caused by sequencing depth. We did not regress out other sources of variances considering they might be users’ research interests. Instead, we used the uGT to store all factors that might cause batch effects so users can choose to remove their effects in downstream analyses according to specific research purposes after retrieving the data from hECA. The normalization processing provides the general-purpose data correction that guarantees the usability of the assembled data.

We conducted many experiments to validate the quality and usability of the assembled data in hECA. We present four example experiments focusing on organ, cell type, and two diseases. The analysis tasks are automatic annotation (label transfer) and finding differentially expressed genes (DEGs).

### Example organ study: using hECA heart data for label transfer

Cell type annotation for a group of cells sampled from a certain organ is the most common use case of label transfer. It can give users a quick overview of the composition of their query cells. We trained an automatic cell type annotation model with hECA data to predict cell type labels of query data not included in hECA. We used accuracy to assess the predicting power of hECA data as label references and to prove the data quality.

We selected data of 160,775 adult heart cells in hECA v1.0 as the reference to train a SingleR (Aran et al., 2019) model for annotation. The query datasets are two recently published human heart single-cell datasets that have not yet been included in hECA, containing 451,513 and 262,003 adult human heart cells, respectively (Litviňuková et al., 2020; Tucker et al., 2020). We mapped the cell type annotations of the reference data and query data onto heart cell types of uHAF framework to unify the different annotation granularities in the original studies. We assessed the performance with a confusion matrix, including accuracy and Kappa score. The results on the two query datasets both reached accuracies over 0.9 (Table S8), indicating the predicting power of hECA assembled data as label reference. We also conducted the same experiments on data of different batches and batch-corrected data with other popular methods. Results showed hECA data performed best on one query dataset (Figure S8). More detailed results of these experiments are provided in the Supplemental experiments in STAR Methods.

### Example cell type study: using hECA neuron data for label transfer

This example corresponds to scenarios where users know the coarse cell type of a group of cells (nodes near the root of uHAF), but need to annotate their finer cell types. We selected all 185,419 cells annotated as neurons or their subtypes in hECA as the reference dataset. The query dataset was the PsychENCODE dataset containing 27,412 cells from prefrontal cortex samples (Wang et al., 2018). We assessed the label transfer accuracy and DEGs of cell types to prove data quality.

The cell type annotations of query cells were in a different label system with uHAF annotation on neurons (Figure S9B), so we manually assigned uHAF cell type labels to the query data with well-known markers as the standard for comparison (Figure S9D). We trained a SingleR model with the hECA neuron reference to annotate the query cells (Figure S9E). UMAP plots show that manually assigned uHAF labels and SingleR labels are consistent in the neuron cells, but differ in the non-neuron cells as the model failed to identify endothelial cells and glial cell types. This was because the query cells included non-neuron cells, but model was trained only on the reference of neuron cell across organs. Except for those non-neuron cells, the overall accuracy of automatic annotation was 0.887 (95% confidence interval [0.882, 0.891]) and Kappa score 0.734 assessed by the confusion matrix.

We then conducted differential expression analysis on the manually and automatically annotated cell types and got two lists of cell-type-specific marker genes. We took the intersection of the two lists and calculated the proportion of common genes in DEGs found by the SingleR model. The coverage was 96.7%, 68.1%, and 62.9% for excitatory neurons, VIP inhibitory neurons, and PV inhibitory neurons, respectively. If we filter out the non-neuron cells in the reference, the latter two coverage raised to 78.9% and 81.9% (Table S9). These results confirmed the reliability of hECA-referenced annotation.

### Example disease study: using hECA data as normal control for cancer study

Using data of the healthy cells in hECA as normal controls for disease studies are a natural application scenario. We designed two example experiments to show how this can be done with hECA data.

The first experiment was on a lung cancer study. Kim et al. published a scRNA-seq study that collected cells from primary and metastatic lung adenocarcinoma (LUAD), and para-tumor normal lung tissues (Kim et al., 2020). The original study analyzed disease and normal cells. We did this experiment to show that if the normal lung tissue data were absent in that study, hECA could fill in the gap for its major findings.

The original work built a single-cell landscape of normal lung and LUAD in primary or different metastatic locations. hECA contains 54,615 well-annotated lung cells covering all major cell types reported in that work. Marker genes used for identifying cell types in their work also showed cell-type-specific expression in hECA data (Figure S10). A major discovery in the original work was that the epithelial cells in normal lung and primary tumor formed a 3-state trajectory. We replaced their epithelial cells from the normal lung with those from hECA, and identified a 3-state trajectory with Monocle2 (Qiu et al., 2017a; Qiu et al., 2017b) (Figure S11). The changes in sample origin proportions and cell type proportions between the 3 states were also similar, although some differences existed, such as the proportion of AT2 and ciliated cells in state 1 (Figure S11). The differences may be caused by differences in sampled abundances of these cell types between the para-tumor samples in the original study and the healthy human lung samples collected in hECA.

### Example disease study: using hECA for label transfer and normal control in a COVID-19 study

The second experiment was on a COVID-19 study. We experimented with hECA lung immune cells as normal control to perform label transfer and DEG analysis for a COVID-19 study. The design of the label transfer experiments is listed in Figure S12A. We collected lung immune cells from hECA by selecting CD45^+^ cells in the lung (Figure S12B). The hECA-assembled cells had three independent sources and formed three batches: batch 1 data were generated using Microwell-seq, and batches 2 and 3 were generated using 10x Genomics. The disease data were bronchoalveolar lavage fluid (BALF) sampled from patients with COVID-19 (Liao et al., 2020). We manually annotated cells in the study with signature genes described in the original study as the standard for comparison (Figure S12C). We conducted four experiments using Seurat label transfer functions to classify the query COVID-19 study cells using different references. Results showed similar performances using hECA data with the baseline single-batch experiments (Figures S12D–S12H, details in Supplemental experiments in STAR Methods), indicating that the assembled data in hECA are reliable to support label transferring.

We further conducted DEG experiments to study the use of the normalized data in hECA. The aim was to find DEGs between the HC (healthy control) group and the S (Severe) group in the data (Figure S12I). We used the DEGs identified on macrophages, dendritic cells, CD8 T cells, and NK cells between the HC and S groups in the original study as the baseline. We did DEG analyses by replacing the original HC cells with healthy cells extracted from hECA data using different batch-effect correction strategies and evaluated the consistency between the found DEGs (Supplemental experiments in STAR Methods). The COVID-19 study and the studies from which hECA collected data were with very different experiment designs; it can be expected that many detected DEGs changed when HC data changed (Figure S12J). We observed that the recovery of the original DEG lists was poor if the popular way of batch-effect correction was used for hECA data from different batches. The hECA-normalized multi-batch HC data performed almost the same as the single-batch HC data with the same technical platform as the COVID-19 data; both are the best for all cell types. We also observed that the enriched pathways of the DEGs found with hECA HC data agreed with the original HC data (Figures S12K and S12L). These observations showed that the hECA-assembled data from multiple sources could be reliably used as health controls. If the sample size allows, it can be better to use only data generated from the same sequencing platform with the disease data.

The experiments on LUAD and COVID-19 data confirmed the quality of the assembled data in hECA and also showed a promising way to use the atlas data in disease studies. The number of cells collected in current hECA v1.0 for many organs is still tiny. With the continuous inclusion of more data for all organs, the assembled cell atlas will better perform the role of a general healthy reference for future disease cell studies.

### *In data* cell sorting enables comprehensive virtual cell experiments as a new research paradigm

Cell sorting is a basic experimental technique in cell biology. We introduced in hECA an “*in data*” cell sorting technique, an innovative virtual cell experiment scheme facilitated by the cell-centric assembly of data of all organs. *In data* cell sorting allows users to select any cell of interest in the atlas according to any features of the cell. When the data in the atlas provide sufficient coverage of all major tissues, organs, and cell types of the human body, the cell-centric assembled cell atlas becomes a virtual human body. To precisely pinpoint the required cells from the virtual body, users can define criteria as combinations of logic expressions, such as desired expression range of one or multiple genes, required organs, tissue origins, developmental stages, donor’s genders, ages, etc. This sorting scheme has higher flexibility, resolution, and finer granularity than traditional cell sorting on *in vivo* or *in vitro* samples. The sorting dimension is not restricted by several surface markers for flow cytometry, but can be extended to precisely measure any number of features. The source materials for the sorting are not restricted by samples collected in one study, but can be extended to all cells with desired properties from all studies in the ensemble atlas. Designing cell experiments becomes a matter of writing a computer code of logic expression for searching hECA. This opens the new paradigm in cell biology: *in data* cell sorting followed by *in silico* computational experiments. This “*in data* experiment” paradigm will facilitate scientists to conduct investigations in the data space beyond the limitations of traditional *in vivo* or *in vitro* experiments.

*In data* cell sorting can be implemented on the hECA interactive web interface or using the Python package ECAUGT. Here, we show a simple example of the sorting: to sort for all T cells in the heart with the normalized expression of gene PTPRC greater than 0.5 and that of CD3D or CD3E greater than 0.5, users can simply type the logic expression in python: rows_to_get = ECAUGT.query_cells("organ = = Heart && cell_type = = T cell", include_children = True) gene_condition = ECAUGT.seq2filter("PTPRC >0.5 && (CD3D>=0.5 || CD3E>=0.5)") ECAUGT.get_columnsbycell_para(rows_to_get = rows_to_get, cols_to_get = ['PTPRC', 'CD3E', 'CD3D'], col_filter = gene_condition)

hECA will return the selection results (of 1,523 cells in the current version) in about 4 s. In this example, considering possible noises and numeric issues, we used 0.5 of the normalized expression value as the threshold for “non-zero” expression instead of using “expression>0”. Users are free to try other thresholds and explore their effects on selecting cells using the online visualizations.

This example shows the logical clarity, convenience, and efficiency of *in data* cell sorting. By contrast, the typical cell sorting workflow composed of multiple filtering steps is more complicated. To obtain the regulatory T cells (Treg) from a certain type of human tissue sample, a researcher needs to use the marker protein PTPRC (also known as CD45) to distinguish immune cells (PTPRC+) from other lineages of cells (PTPRC-), use CD3 to select the T cells (PTPRC+ and CD3^+^) from the PTPRC+ cells, and then use CD4, IL2RA (also known as CD25), and FoxP3 markers to filter out other T cells and get the Treg cells. The types of cells can be selected depending on the availability and identifiability of surface markers of the cells under study, and the discriminating power of the flow cytometry technology. This sorting practice is much lengthier and more time-consuming than the *in data* sorting. And *in data* sorting can apply many selection criteria that may not be possible for flow cytometry. With the growing coverage of hECA, researchers can conduct all kinds of pre-experiments with *in data* cell sorting to accelerate the research loop.

Another advantage of *in data* cell sorting is swift multi-step iteration. Users can jump back and forth in sorting steps to make comparisons for optimal results. They can adjust sorting criteria based on analysis of previous steps, without waiting for another experiment loop. For users to have a quick overview of sorted cells, we provided a real-time analysis function on the web interface. The real-time analysis includes the following properties of the selected cell group: 1) cell type composition in all and every organ, 2) expression distribution of interested gene across cell types and organs, and 3) “FACS-like” plots to show relative expression levels between any two interested genes. Users can conduct the next step of cell sorting based on real-time analysis results, without the trouble of downloading and locally analyzing the whole dataset. We provided five examples of utilizing *in data* cell sorting, three of them are done with the web interface, and the other two are shown in ECAUGT with vignettes and detailed explanations (see STAR Methods).

We conducted two application examples on leveraging the potential of *in data* cell sorting: 1) agile construction of atlases of particular cell types and 2) off-target prediction of targeted therapy. These cases demonstrated in detail how hECA could be used to conduct comprehensive studies of cells across the human body in an unprecedented way.

### Case study 1: agile construction of a draft T cell metabolic landscape

In the first case example, we built a draft T cell sub-atlas to show the power of hECA in the agile construction of cell landscapes across studies and across organs. This case also shows how to compare the metabolic activity heterogeneities between different organs/cell types in a high-throughput way from the public data.

T lymphocyte is an essential cell type in the human immune system. They adapt to multifarious microenvironments as they circulate through or reside in the human body. Their differentiation, activation, and quiescence are regulated by diverse metabolites in the local microenvironment (Buck et al., 2015; Chapman et al., 2020; Shyer et al., 2020; Yin et al., 2019). Recent studies reported that microbial bile acid metabolites promoted the generation of regulatory T cells in the intestine, which is associated with inflammatory bowel disease (IBD) (Campbell et al., 2020; Hang et al., 2019; Song et al., 2020), suggesting that targeting metabolic pathways of T cell activation and differentiation may improve therapeutic outcomes of patients with IBD (Li et al., 2021). A comprehensive survey of the metabolism of T cells across multiple organs is crucial for better understanding intrinsic responses of T cells to microenvironment changes, but *in vivo* or *in vitro* experiments on multiple organs are not easy. Xiao et al. proposed a computational pipeline to study the metabolic landscape of cells from single-cell transcriptomic data (Xiao et al., 2019). The cell-centric assembly of cells of all types in all organs in hECA allowed us to conduct *in data* study on T cell metabolism across all organs, instead of searching through datasets scattered in the literature.

**Figure 2:**
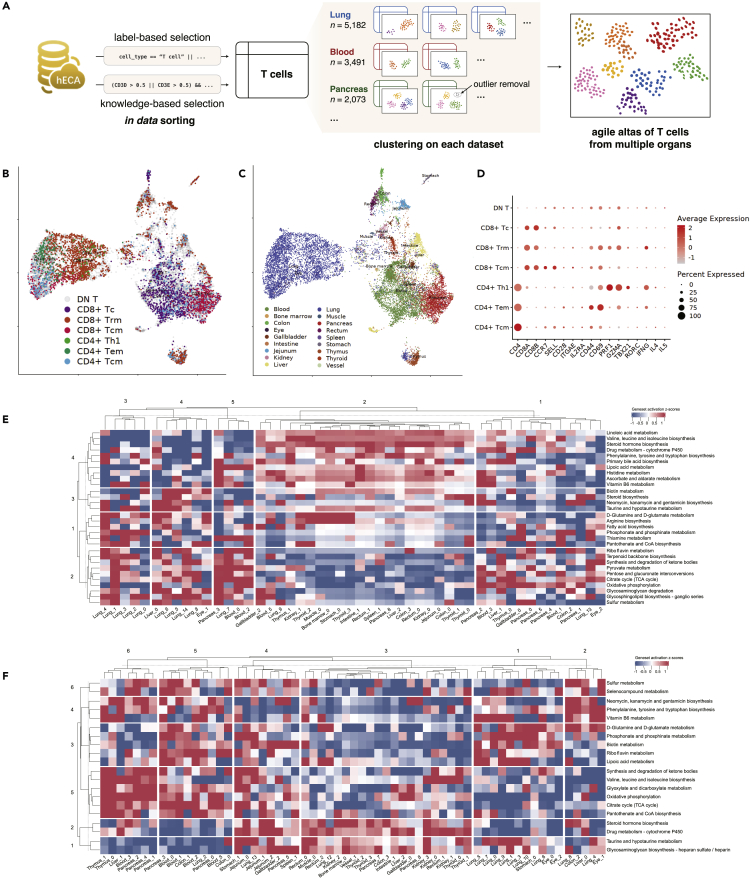
The agile construction of a draft T-cell metabolic landscape across multiple organs from hECA (A) Workflow of the *in data* cell sorting from hECA to build the agile T cell atlas. (B) Subtypes of selected T cells are displayed on UMAP. DN T: Double negative T cell, CD8^+^ Tc: CD8^+^ Cytotoxic T cell, CD8^+^ Trm: CD8^+^ resident memory T cell, CD4^+^ Th1: CD4^+^ T helper cell type 1, CD4^+^ Tem: CD4^+^ effector memory T cell, CD4^+^ Tcm: CD4^+^ central memory T cell. (C) Organ origins of selected T cells organ origin displayed on UMAP. (D) Gene expression signatures of the identified T cell subtypes. The color bar represents average expression level of cell type related markers with colors gray to red indicating expression low to high. The dot size represents percentage of cells expressing the marker within subtypes. (E–F) Heatmaps showing z-scores of activity scores of major metabolic pathways of the T cell subtypes in multiple organs. (E) for CD4^+^ T cells and (F) for CD8^+^ T cells. Each row in the heatmap corresponds to one selected term in the KEGG metabolism pathway database, and each column corresponds to one T cell subcluster. See also Figures S1 and S2.

Using ECAUGT, we first sorted all cells in uGT with the label “T cell” and associated names (such as “CD4 T cell”, “CD8 T cell”, “Activated T cell”, etc.) across all organs (Figure S1A). To include cells that might be annotated to other cell types, we also searched for cells with normalized expression values of PTPRC, CD3D, or CD3E greater than 0.5 across all organs (Figures S1B and S1C). Then, we filtered the cells by the expression of a list of negative markers such as COL1A1 and CD79A (the complete list is provided in Table S4). We conducted clustering analysis on cells from the same organs, and obtained a series of candidate clusters in each organ (Figure 2A). We removed clusters with low expression levels of CD3D, CD3E, or CD3G as they are unlikely to be T cells. After these steps in hECA, we built an agile cell atlas of T cells across 18 organs (lung, pancreas, blood, liver, muscle, thymus, jejunum, rectum, colon, kidney, gallbladder, stomach, thyroid, intestine, spleen, bone marrow, eye, and vessel). The details of these steps are provided in STAR Methods, and the codes are given at http://eca.xglab.tech/ecaugt/T_cell_analysis_with_in-data_cell_sorting.html#t-cell-analysis-label.

The following experiments are downstream analyses performed out of hECA to prove the viability of the constructed T cell atlas. To assign accurate annotations to the cells in the constructed T cell atlas, we performed hierarchical clustering using signature genes CD4, CD8A, and CD8B, and divided the cells into 6 subgroups of 3 major groups (Figure S2A). The three major groups are CD4^+^, CD8^+^, and double-negative (CD4^−^and CD8^−^) T cells (Figure S2B). For the CD4^+^ and CD8^+^ groups, we further annotated the cells as resident memory T cells, central memory T cells, effector memory T cells, naive T cells, cytotoxic T cells, etc., according to the positive markers listed in Table S5. Figures 2B and 2C show the UMAP of the CD4^+^ and CD8^+^ T cells with the subtype annotations and the cells' organ origin, respectively. Figure 2D shows the gene expression signatures of the identified T cell subtypes. For the double-negative cluster, we marked them as “T cells” without further analysis as there might be cells false negatives in CD4 or CD8 expression due to possible dropout events in scRNA-seq data.

For a sketchy study on the metabolic landscape of T cells across multiple organs, we evaluated each cell’s metabolic activity scores with GSVA, which produced comparable values across multiple clusters or datasets and alleviated possible batch effects in the data from multiple sources (Hänzelmann et al., 2013). The genes of the metabolic pathways were derived from KEGG (Kanehisa et al., 2020) and Xiao et al.’s work (Xiao et al., 2019). Figures 2E and 2F show heatmaps of the obtained draft metabolic landscape of T cells of their activity scores of all major metabolic pathways across the human body. Such landscapes can help to reveal different metabolic patterns across organs. For example, we found organ-level metabolic variations in the lungs from the metabolic activities of organ-level CD4^+^ T cell clusters in Figure 2E and those of the organ-level CD8^+^ T cell clusters in Figure 2F. For CD4^+^ T cells, we observed lung-enriched metabolic pathway activations in the pathways of riboflavin metabolism, terpenoid backbone biosynthesis, TCA cycle, oxidative phosphorylation, sulfur metabolism, and D-Glutamine and D-glutamate metabolism (row blocks 1 & 2 of the lung-origin T cell clusters in Figure 2E). Similar enrichments can also be observed in the lung-origin CD8^+^ T cell clusters in Figure 2F.

These observations deserve further investigation. They showcased the potential of cross-organ *in data* cell experiments enabled by hECA, which are otherwise hard to conduct in traditional experiment settings. The draft T cell atlas constructed with data from multiple sources assembled in hECA also shows that the standardized preprocessing and normalization in hECA ensured the reliability of the data.

### Case study 2: *in data* discovery of side effects in targeted therapy

In the second case example, we utilized *in data* cell sorting to investigate possible off-target effects in cancer therapy. This case study shows hECA’s potential application in disease studies and virtual drug experiments.

A significant part (∼97%) of cancer drugs tested in clinical trials failed to get approval from FDA, mainly due to their insufficient efficacy or unexpected toxicities to organs where drugs were not designed to take effect (Lin et al., 2019). Off-target effects are usually not easy to observe in animal models. Prediction of cellular toxicities across the whole body can significantly reduce improper clinical trials and increase the efficiency of new drugs discoveries. This is a typical scenario where we should conduct *in data* experiment on the virtual human body of cells to test drugs before clinical trials on human patients.

In previous research, computational investigation of off-target effects or neurotoxicity effects of targeted therapy took multiple steps. Researchers first chose a group of organs as suspects of side effects based on existing knowledge. They needed to review the literature to search for single-cell datasets in which cells in the suspected organs have highly expressed target genes of the candidate drug. Then they would evaluate the effect of the drug on the phenotype of these cells and therefore on the phenotype of the organs. This is a typical setting of traditional “meta-analysis”. Parker et al. found that CD19^+^ mural cells in the human brain were potential off-tumor targets of CAR-T therapy in this way (Parker et al., 2020). They first noticed from previous literature that CD19 CAR-T therapy could introduce neurologic adverse reactions. Then, they collected 3 single-cell datasets of the human brain: prefrontal cortex (Zhong et al., 2018), forebrain (La Manno et al., 2018), and ventral forebrain (La Manno et al., 2016). After reprocessing each dataset, they manually annotated cells by comparing highly enriched genes to known cell type markers. They observed on the UMAP a small population of cells in the first dataset expressing both CD19 and CD248 (a marker for mural cells). They further identified these cells as pericytes and verified them in all three datasets. This type of meta-analysis depends much on the existing hints or guesses on possible off-target organs and involves heavy efforts in data collection and reprocessing.

**Figure 3:**
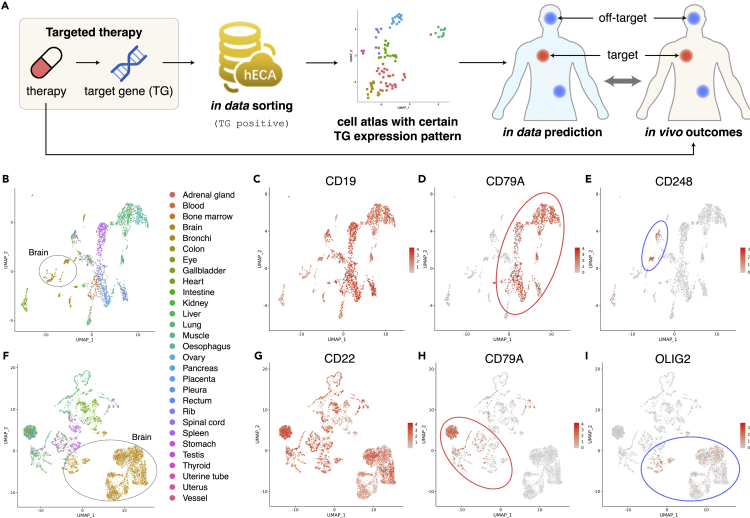
*In data* experiments with hECA facilitating discoveries of side effects of targeted drugs (A) The diagram of using *in data* cell sorting to predict targets and off-targets of targeted therapy. Red dots and blue dots in the human body represent the intended target sites and side-effect sites, respectively. The red and blue dots in the UMAP represent the intended treatment cells and side-effect cells, respectively. (B) Visualization of CD19^+^ cells (expression>0.1) in UMAP, colored by organ origins of cells. CD19 is the target gene of the targeted therapy. (C) Visualization of CD19 expression levels of those CD19^+^ cells. (D) Visualization of CD79A expression levels of those CD19^+^ cells. CD79A is a marker for B cells. (E) Visualization of CD248 expression levels of those CD19^+^ cells. CD248 is a marker for pericytes. (F) Visualization of CD22^+^ (expression>0.1) cells in UMAP, colored by organ origin of cells. CD22 is the target gene of the targeted therapy. (G) Visualization of CD22 expression levels of those CD19^+^ cells. (H) Visualization of CD79A expression levels of those CD22^+^ cells. CD79A is a marker for B cells. (I) Visualization of OLIG2 expression levels of those CD22^+^ cells. OLIG2 is a marker for oligodendrocytes. The color bars in (C–E) represent expression levels of CD19, CD79A, and CD248, and the color bars in (G–I) represent expression levels of CD22, CD79A, and OLIG2, respectively, with colors gray to red indicating expression low to high. The red and blue ellipses in (D–E) and (H–I) line out the target cells and off-target cells, respectively. See also Figures S3 and S4.

We followed the example of Parker’s work (Parker et al., 2020) to study the possible off-target effects of CAR-T therapy in a more automatic way using hECA. CD19 is a common target of CAR-T therapy in treating B cell lymphoma (Wei et al., 2019). Neurological toxicity is one of the major known side effects (Rubin et al., 2019). To study why this toxicity occurs and whether other organs might also be affected by CAR-T therapy, we used a filtering criterion on CD19 expression for *in data* cell sorting in hECA. Totally, 2,566 CD19^+^ cells passed the filter (Figure 3B). This therapy aims to target malignant B cells for curing lymphoma. But B cells and plasma B cells only compose ∼53% of the selected CD19^+^ cells (Figure 3C, S4, Table S6). The other cells in the selected group include endothelial cells, microglia and neurons in the brain, cardiomyocytes, fibroblasts in the heart and lung, enterocytes in the rectum, etc. (Figure 3D, S4 and Table S6). They all have the potential of suffering from off-targets of the therapy. This result explains why encephalopathy was often observed, and cells constructing vessels were targeted by the drug (Parker et al., 2020). Our results also suggest that there are possible toxicities on the circulatory system and digestive system, which can also be validated by reports in the literature (Yáñez et al., 2019).

CD22 is another popular target when designing CAR-T therapy for lymphoma (Wei et al., 2019). Similarly, we used *in data* cell sorting in hECA and obtained 8,724 cells with CD22 expressed (Figure 3E). In addition to B cells (Figure 3F, S5 and Table S7), this group contains oligodendrocytes and excitatory neurons in the brain, cardiomyocytes and fibroblasts in the heart, macrophage, mast cells, and monocytes in the lung, neutrophils in the testis, etc. (Figure 3G, S5 and Table S7). These observations provide significant clues for systematic investigation of the potential side effects of targeted therapy.

Detailed descriptions of the procedures of this case study are given in STAR Methods, and the codes are given at http://eca.xglab.tech/ecaugt/ECAUGT_CD19.html#ecaugt-cd19-label. This study provided an example of a systematic approach to conducting meta-analysis with *in data* cell sorting in a more efficient and effective way based on the cell-centric assembly of massive single-cell data in hECA. For any specific target gene, cells that highly express the gene can be found through *in data* cell sorting, no matter which original datasets the cells are from. A profile of the cellular distribution of all major human organs that contain the found cells can be built, which highlights suspected organs that might be the off-targets of the drug. Detailed analyses can be further applied to the possible effects of the drug on the phenotypes of the cells by checking on the consequences of the expression change of the target gene on downstream gene expression, signaling pathways, metabolisms, interactions with other cells, etc. Quantitative analysis then can be applied to the cell compositions and cell-cell interactions in the suspected organs to evaluate the possible physiology or pathology effects. Users can adopt this approach and apply it to any target cell type they want to investigate. With more cells and richer omics features assembled into the atlas, this type of investigation will lead to a new paradigm of “*in data* clinical trial” for a new therapy that will significantly reduce the risk and cost of the real-patient clinical trial, and increase the efficiency of drug development.

### Quantitative portraiture of genes, cell types, and organs

The above sections illustrated how users could explore and exploit hECA with the flexible and cell-centric *in data* cell sorting engine. To better describe whole vivid pictures of the biological entities in hECA, we developed a “quantitative portraiture” system. The system contains a set of quantitative portraits of the biological entities, including organs, cell types, and genes for all quantifiable characteristics at multiple angles. We portrayed them in the web interface at all possible levels and aspects so that users can get a comprehensive understanding of the whole system, all elements in it, and their relationships. This is an upgraded approach from the current approach of using “snapshots” of marker genes to describe a cell type. The current version portrayed 38 organs, 146 cell types, and 43,878 genes in hECA v1.0 with the currently available data. With the growing coverage and quality of data assembled into hECA in the future, the portraiture framework will lead to “holographic” macroscopic and microscopic views of genes, cells, tissues, and organs of the human body.

In gene portraits, we showed the expression distribution of a gene in each selected organ or cell type, providing a quick overview and organ-wise or cell type-wise comparison of genes of interest. We also included basic information about the gene, links to GeneCard, NCBI, Ensemble, and Wikigene pages of the gene. The design of gene portraits borrowed the idea from the “gene skyline” of ImmGen (http://rstats.immgen.org/Skyline/skyline.html), a project that collects immunological data and profile gene expression signatures. In the portrait page of gene *PTPRC* (Figure S5), for example, the basic information of the gene is firstly shown, including the gene’s full name *Protein Tyrosine Phosphatase Receptor Type C*, some of the aliases, its location on the chromosome, etc. A panel “*Known as markers of*” provides information about cell types in which the gene is highly expressed. Users can browse the distribution of the gene’s expression level, grouped by the uHAF organ tree or cell type tree. The gene portraits in hECA present several major improvements compared with the gene skyline. Firstly, the distribution is provided for each gene in each cell type or organ instead of only the mean value of expression level. Besides the function of exhibiting relative expression strength between cell groups, expression distributions show more information like the percentage of cells that express the gene, or heterogeneity within a cell type which may indicate potential sub-types. Secondly, hECA gene portraits cover a wider breadth of cell types, while the data of gene skyline were restricted to immune cells. Furthermore, hECA portraits are based on the uHAF annotation. This allows the portraits to be updated timely with the expansion of uHAF when more data are assembled.

hECA cell type portraits include the organ origins of a certain cell type, marker genes in the cell type, view of the cell type in embedding space, and the position of the cell type in the uHAF tree (Figure S6). A cell type is mainly characterized by two types of information: organs containing the cell type and the expression patterns of genes specific to the cell type. hECA v1.0 portrayed 146 of the 416 cell types organized by the hECA hierarchy with the current data availability. On the hECA website, users can type in the name to search for a cell type or click along the tree of cell types to display the cell type portrait. It includes the distribution of the cell type across organs, shown as the number of cells of this type collected in the organs, the list of marker genes with their characteristic expression ranges in the cell type, and a 2D PCA, UMAP, or DensMAP visualization (McInnes and Healy, 2018; Narayan et al., 2021; Pearson, 1901) of the cells colored by the organ of the cells or the expression of a particular gene in the cells.

hECA organ portraits include organs’ cell type composition, an embedding view of cell types in the organ, and a tree view of its position in uHAF (Figure S7). An organ is usually characterized by its anatomic and physiological features, but the full portraiture of an organ should include its complete cellular and molecular features at multiple resolutions. The basic cellular information is the relative composition of cell types in the organ and in its different anatomical parts. The basic molecular information is the gene expression patterns in the organ as a whole and its different parts, spatial locations, and at different physiological statuses. In the embedding viewer, we show the feature map of each gene in 2D visualization, showing the relationship between certain genes, cell types, and organs. The current coverage and quality of the data are still far from fully characterizing the entities in an unbiased manner. Therefore, current portraits can only reflect information in the collected data rather than the complete biological picture. But the portraiture framework provides a comprehensive approach leading to the whole picture when more and more data are assembled into hECA.

It should be noted that most current single-cell sequencing technologies undergo cell selection before sequencing. For selected cells, the sampling efficiencies for different cell types are also not uniform (Baran-Gale et al., 2017; Phipson et al., 2017; Tung et al., 2017). Many technical reasons may cause biases in the measured gene expression values even in the same experiment, let alone across different experiments (Chen and Zheng, 2018; Miao et al., 2018; Miao and Zhang, 2016; Soneson and Robinson, 2018). Therefore, it is unrealistic to expect the current portraits of genes, cell types, or organs to be of full fidelity given the currently available technology and data. With this limitation in mind, users can use portraits to explore the state-of-the-art information of the objects. The hECA quantitative portraiture system provides a framework presenting the complete information of biological entities, and sets a goal for future ideal cell atlases.

### Customized reference creation for automatic cell type classification

Every cell in hECA has standard identity labels chosen from the uHAF. Users can transfer these identity labels to their own datasets with published or in-house cell type classifiers. Many computational tools for automated cell type identification have emerged, as described in (Pasquini et al., 2021). These classification methods rely on a good selection of reference datasets to perform good label transfer because different cell compositions in the reference data may lead to differences in annotation results. For instance, when studying hematopoietic development, training a classifier with a reference containing only hemocytes will reduce misclassification.

In hECA, the *in data* cell sorting web and programming interfaces can help users create customized references using flexible creation criteria. We provided a list of pre-created reference datasets organized by organs, which is available under the uHAF menu in hECA at http://eca.xglab.tech/ (Home > uHAF Cells > Automatic Annotation). We did two label transfer experiments at the organ level and cell type level as examples to illustrate the use of hECA references for automatic cell type classification and quantitatively assess the performances. Both experiments showed an accuracy of around 0.9 (0.954 and 0.904 for different heart datasets; 0.887 for neurons), proving the predicting power of hECA-sorted data as label reference; the details and results are provided in the above Data Quality Validation session. Users can follow these examples to compose reference datasets according to the need of their particular studies. It should be kept in mind that the current curated references from hECA may not be complete in the cell type composition for some organs due to the insufficient data coverage and possible biased sampling in the current data. The references will be more complete with the continued assembly of new data into the atlas.

## Discussion

We presented hECA, a cell-centric-assembled human cell atlas based on the collection of data scattered in the literature. hECA was empowered by a unified informatics framework providing structured indexes and a combinatorial searching facility. The cell-centric assembly provides three novel applications of the ensemble atlas that could be difficult for file-centric data collections: 1) a new experiment paradigm “*in data*” cell sorting that enables efficient selection of cells across the whole body that meet combinations of multiple logic conditions, 2) a “quantitative portraiture” system for holographic characterization of biological entities, and 3) a customizable reference generation function for automatic annotation of users’ query cells. These are based on three technologies in the unified informatics framework we developed for cell atlas assembly: uGT, uHAF, and ECAUGT. The current assembly of ∼1 million cells of 38 organs in hECA v1.0 only provided a low-coverage atlas. But example applications have already demonstrated the revolution that such a cell-centric ensemble cell atlas can bring to biomedical research beyond the possibility of individual single-cell studies or file-centric atlas collections.

There have been several other efforts for gathering, collecting, and archiving single-cell data. Those “data integrations” are at the dataset level rather than cell level: Data of cells from different studies and sub-studies are archived as separated files rather than merged into a single database; databases are used to manage or index the metadata of the datasets instead of the individual cells. The typical way to use those resources is to find specific datasets from the list and download the corresponding data files to users’ local computers for in-house analyses. They provided useful resources for many studies. But it is not convenient or efficient if users need to utilize data across multiple datasets in a more comprehensive manner. Tasks such as evaluating the expression of a particular gene among multiple organs or studying cellular emigrant routes need researchers to process dozens of datasets separately. These tasks require cell-centric assembly of data across studies and datasets. There has been no such reported effort yet for assembling massive single-cell data of multiple studies into a unified repository. The question of possible underlying information structures to organize and annotate all cells in an atlas has not been sufficiently studied. The unified informatics framework we developed in hECA provides a promising solution for the cell-centric assembly of cell atlas with existing data.

Although the number of cells in hECA v1.0 is still small and the coverage of organs and cell types is very limited, case studies using this primary version already showed the advantage of cell-centric atlas assembly, especially the power of *in data* experiments enabled by the assembly. The customizable annotation reference shows the other way of utilizing *in data* cell sorting. The proposed gene, cell type, and organ portraitures provide a powerful framework for characterizing the complete information of biological entities in a quantitative manner. Up to now, all single-cell data that have been ever generated for human cells are still only a tiny fraction of all human cells, and the data are also under the influence of multiple types of noises and biases. Therefore, the current portraits can only reveal properties of the collected data but cannot be expected of full fidelity for the underlying biology. However, with the currently available data, users can already use these portraits as handy tools for exploring the properties of genes, cell types, and organs from a more complete view than traditional views. With the rapid advancement in data depth, coverage, and quality, the portraits will provide multi-scale holographic views of all biological entities in the human body. We have developed strategies and technologies to continue assembling new data into hECA from all available sources. When other large-scale cell atlases are being constructed, hECA will be a complementary system to those atlases with the unique features we invented based on the cell-centric assembly.

Several upstream processing issues are crucial for the construction of cell atlases, such as normalization and correction for possible batch effects. Non-uniform sampling of cells and expressed genes is another issue that may poison global analyses of atlas data. In building hECA, we followed the currently widely accepted protocols and best practices for the upstream processing of collected data. Especially, possible systematic differences between data from different studies are a major concern in integrating data. Such differences can make the expression values of genes not comparable among different studies. We evaluated the well-received batch-effect correction methods. They were all designed for specific downstream analysis tasks, typically for cells of the same organ rather than across all organs of the human body. Most of them aligned cells from different batches in some latent representation space, and were not designed for converting the original expression values to adjusted expression values with preserved biological meanings. The purpose of building the assembled cell atlas is to provide a general reference for all possible future uses. Therefore, we chose to apply the basic batch-correction step of normalizing gene expression values in different batches by the library sizes. This normalization is important for all types of downstream analyses, and it keeps the adjusted expression data biologically interpretable and comparable. All example experiments have shown that such basic normalization safeguarded the usability and reliability of the assembled data. For studies that need more specific batch-effect correction on data retrieved from hECA, the rich meta-data accompanying the retrieved cells allow users to apply an extra correction for factors that are regarded as batch-effects in their studies. For the convenience of some users, we also provided downloadable data files that have been batched-corrected for each organ using existing methods (STAR Methods).

The work on data integration continues in the single-cell community and these methods will be incorporated into hECA if proven beneficial, but regardless, a cell-centric framework to query the data is very much required to enable researchers fully utilize atlases of this size. From the case examples, we can see that *in data* cell sorting on the assembled data can reveal important organ-specific patterns and can help to discover organs that are more prone to side effects of targeted therapy. It can be expected that in the future, with deeper coverage of cells from all organs, better quality of original data, and more measurable features for cells, the cell-centric assembly of hECA will grow into a rich virtual human body that enables more advanced “*in data* experiments” to answer questions that can hardly be investigated *in vivo* or *in vitro*, and enables multiscale holographic quantitative portraitures of all biological entities about the human body*.*

### Limitations of the study

The number of cells collected in hECA v1.0 was still small. Some of the data we collected was with earlier single-cell sequencing technologies, so the data qualities are not equal. These limitations on the data coverage and qualities should be kept in mind when using the data. We have developed strategies and technologies to integrate data from other comprehensive datasets and new datasets in the future. We will continue improving and updating hECA in future versions by assembling more high-quality data.

The batch-effect issue in data integration is one of the biggest computational challenges of single-cell expression study. We took the basic correction procedure in building hECA v1.0 by normalizing data from different batches with the library sizes. In this way, the corrected gene expression values kept their biological meanings and are comparable. However, there could be variances due to non-biological factors that cannot be corrected in this way. We provided the convenience for users to do extra corrections according to the need of their downstream analyses of the data retrieved from hECA. The field is developing quickly in both single-cell sequencing technologies and computational methods. We will keep improving the general-purpose data integration in hECA by developing or incorporating new techniques. We will include more customizable tools for users’ special-purpose data integration tasks in future versions.

## STAR★Methods

### Key resources table

**Table:** 

RESOURCE	SOURCE	IDENTIFIER
**Deposited data**		

hECA database	This paper	http://eca.xglab.tech/
ECAUGT	This paper	https://pypi.org/project/ECAUGT/
hECA database (the extra version)	This paper	http://eca.xglab.tech/#/resources; https://ngdc.cncb.ac.cn/omix/release/OMIX001042

**Software and algorithms**		

Seurat	(Stuart et al., 2019)	https://github.com/satijalab/seurat
SingleR	(Aran et al., 2019)	https://www.bioconductor.org/packages/release/bioc/html/SingleR.html
Scanorama	(Hie et al., 2018)	https://github.com/brianhie/scanorama
Harmony	(Korsunsky et al., 2019)	https://github.com/immunogenomics/harmony
BiocNeighbors	-	https://bioconductor.org/packages/release/bioc/html/BiocNeighbors.html
Scanpy	(Wolf et al., 2018)	https://scanpy.readthedocs.io/en/stable/index.html
Monocle2	(Qiu et al., 2017a; Qiu et al., 2017b)	http://cole-trapnell-lab.github.io/monocle-release/
Enrichr	(Xie et al., 2021)	https://maayanlab.cloud/Enrichr/
WikiPathway	(Martens et al., 2020)	https://www.wikipathways.org
biomaRt	(Smedley et al., 2009)	https://bioconductor.org/packages/release/bioc/html/biomaRt.html
Plotly	-	https://github.com/plotly/plotly.py
KEGG	(Kanehisa et al., 2020)	https://www.genome.jp/kegg/
ontologyIndex	(Greene et al., 2017)	https://cran.r-project.org/web/packages/ontologyIndex/index.html
GSVA	(Hänzelmann et al., 2013)	https://www.bioconductor.org/packages/release/bioc/html/GSVA.html

**Other**		

uHAF macroscope ontology	This paper	https://github.com/XuegongLab/hECA
uHAF microscope ontology	This paper	https://github.com/XuegongLab/hECA
uHAF macro-micro map	This paper	https://github.com/XuegongLab/hECA
uHAF marker reference	This paper	https://github.com/XuegongLab/hECA
HGNC gene symbol list	This paper	https://github.com/XuegongLab/hECA
GeneSymbolUniform toolkit	This paper	https://github.com/XuegongLab/hECA
CellMarker	(Zhang et al., 2018)	http://biocc.hrbmu.edu.cn/CellMarker/
The Human Protein Atlas	(Uhlén et al., 2015)	https://www.proteinatlas.org/
PanglaoDB	(Franzén et al., 2019)	https://panglaodb.se

### Resource availability

#### Lead contact

Further information and requests for resources should be directed to and will be fulfilled by the Lead Contact, Xuegong Zhang (zhangxg@tsinghua.edu.cn).

#### Materials availability

This study did not generate new unique reagents.

### Experimental model and subject details

References to original studies that generated the single-cell transcriptome datasets analyzed in this work can be found in Table S1.

### Method details

#### Dataset collection

In the first version of hECA (v1.0), we presented an atlas of 1,093,299 cells from 116 datasets belonging to 21 published studies (Asp et al., 2019; L. Chen et al., 2022a; Cui et al., 2019; Gaublomme et al., 2019; Han et al., 2020; Kinchen et al., 2018; Lake et al., 2018; Lukowski et al., 2019; Madissoon et al., 2019; Menon et al., 2019; Parikh et al., 2019; Plasschaert et al., 2018; Renthal et al., 2018; Sunkin et al., 2013; Venteicher et al., 2017; Vieira Braga et al., 2019; Voigt et al., 2019; Wang et al., 2020a; Wang et al., 2020b; Zhong et al., 2020; Zhong et al., 2018), details provided in Table S1. We designed hECA as an instance for cell-centric assembly of cell atlas by collecting all accessible human single-cell data into a unified atlas, regardless of the technology, platform, researcher, study design, or other factors in data generation. Toward this goal, we selected 20 peer-reviewed studies, preferably studies with high throughput in cell numbers and coverage of multiple healthy organs. These studies covered 38 organs and spanned the developmental stages from fetal to adult. In hECA v1.0, we only include transcriptomic data of healthy donors, but future versions will cover multi-omics data and data of disease samples.

In each of the studies, we collected the expression matrix of every dataset in the study. In addition, we collected all the descriptive information at study level and dataset level, and analyzed the results of the cells in the original papers. They are referred to as metadata in hECA. Metadata includes the following information, if available: sample organ, sample tissue, anatomical region, subregion, donor ID, donor gender, donor age or developmental stage, sequencing technology, and original annotations that are the assigned cell type label of each cell in the original study. The completeness of metadata and annotations vary among datasets according to original studies.

#### Processing of data matrixes

The collected datasets were processed for integration into uGT. The 116 datasets we collected in hECA v1.0 are all single-cell gene expression profiles. Every profile was transformed into a gene-by-cell matrix, with each row representing a gene and each column representing a cell. For those expression values in log scale, we performed the value transformation back to raw values.

To integrate data into uGT, we unified the gene names for all datasets. For datasets identifying genes with Ensembl IDs, we used the R package biomaRt (Smedley et al., 2009) to convert Ensembl ID into gene symbol. Then the gene symbols of different datasets were unified with an in-house built toolkit: we compared gene symbols in the datasets to the list of 43,878 HUGO Gene Nomenclature Committee (HGNC) approved symbols (see “HGNC gene symbol list” in https://github.com/XuegongLab/hECA), all previous, withdrawn and alias symbols were converted into HGNC approved symbols. Genes in the list but not sequenced in any dataset were filled with zeros. After processing, every expression matrix was with 43,878 genes as rows.

For datasets with cell type annotations in the original study, the original annotations were kept and stored in column “original_name” in the uGT. Regardless of the original annotations, we performed clustering analysis and annotation in each dataset with Seurat v3.2 (Stuart et al., 2019). We implemented a standard processing procedure for each dataset: We created a Seurat object from the expression matrix, conducted quality control to filter out genes and cells, selected variable genes, conducted normalization, scaling, dimensional reduction, and cell clustering. The parameters for quality control and cell filtering were determined specifically for each dataset following the original studies or following the tutorial of Seurat. The parameter for cell clustering is determined based on the consistency with the original cell clustering results. Then the analysis pipeline of Seurat was performed to get cell cluster-specific expressed genes. After the quality control, we got 1,093,299 cells from 116 datasets. All codes for processing the data are provided in https://github.com/XuegongLab/hECA/

#### Data normalization and batch adjustment

hECA assembles data from multiple sources and provides sorted data for collective downstream analyses. Normalizing data from different sources to make them comparable is important for the assembly. In data processing, we adopted a library-size-based data adjustment method to normalize data from different batches. The term "batch effect" refers to a mixture of factors that may introduce undesired variations between samples that are supposed to have minor differences. In single-cell RNA sequencing data, many factors may cause "batch effects", including variations from sampling (different donors, body parts, organs or tissue origins, sample treatment protocols, sampling time, etc.), the experiment (different labs, operators, batches, multifaceted cell states such as perturbation, stimulation, activation, etc.) and the sequencing technology (different library sizes, molecular feature detection preferences, etc.). The factors that may introduce batch effect are sophisticated, nonlinear, and nested, and can be far beyond what we’ve mentioned (Luecken et al., 2022). The intension and extension of batch effect are task-specific and not universally agreed upon, depending on the particular biological questions. Researchers with different analysis purposes would choose different pre-processing pipelines and integration methods. Some unwanted variances in one study might be important features in other studies. Therefore, for an atlas designed to be a general reference for all possible downstream tasks, we should avoid over-correcting possible batch effects and leave more space for users to apply task-specific processing after retrieving data from hECA. After a careful study of literature on batch-effect correction and our pilot studies, we chose to use library-size-based data adjustment in normalizing data from different batches so that the data can be used for all types of downstream tasks. For certain types of downstream tasks, extra batch-effect correction may be necessary. After sorting the required cells from hECA, users can check the information in the metadata to identify factors that might be considered as batch effects for their study, and adopt extra processing to correct them before their specific downstream analyses. We conducted several experiments to validate data quality (Figures S8–S12, Tables S8, and S9)

#### An extra version of hECA data with batch-correction

Considering some users may prefer to be able to download data that have been processed with more batch-correction processing, we also provided a downloadable extra version of the hECA data that were processed with an extra step of batch-effect correction. We did a series of comparison experiments on major popular methods, and chose to use the Harmony method (Korsunsky et al., 2019). Harmony is one of the most used integration methods, but it does not provide the corrected matrix. We extended the standard Harmony method by calculating the corrected expression matrix from the vectors in the corrected embedding space with the inverse transformation. We experimented with multiple methods for cross-organ batch-effect correction, but none worked well since there are no shared cell types across some organs. Therefore, we applied the extended Harmony method on cells of the same organ, and did this batch-effect correction separately for each organ. We provided the data thus processed as an extra version of the hECA data for each organ at http://eca.xglab.tech/ (or https://ngdc.cncb.ac.cn/omix/release/OMIX001042).

#### uGT: a unified giant table for assembling cell atlases

To support online "cell-centric" data assembly, we developed a unified giant data table (uGT) to store data from multiple studies into one cloud repository. The unified giant table supports storing high-dimensional omics data and searching cells with dataset-associated attributes (e.g., organ, gender, donor age, study DOI number) and cell-specific features like cell type and $∼10^{4}$ gene expression levels.

The key difference between uGT's NoSQL database and the traditional databases is that uGT used column-based storage layouts for high-dimensional big data. Popular implementations of traditional SQL databases have a rigid width limit for each data item. For example, the limit on the number of columns is 1000 for Oracle™ and 4096 for MySQL™, which has already reached the theoretical upper limit (MySQL, 2021; Oracle, 2021). The number of features of each cell exceeds this limit by several magnitudes. In addition, searching high-dimensional data in row-layout databases is difficult because even if one or two columns are used for data selection, all columns are retrieved by the computer. However, in column-based databases, the column retrieving activity is restricted to the associated columns, which significantly promotes the searching efficiency, although the insertion and update of data become difficult.

With such a design, uGT can store and query almost millions of features of mixed data types for any number of cells if enough storage is given. It can further support more features when features from other omics data are ready to be integrated.

#### Uploading data to uGT

The uGT accepts preprocessed data submission via authorized API. In this version, the data were depth-adjusted and log-normalized and followed one consistent format, ready for uploading. We uploaded 1,093,299 cells to the uGT in total. Every cell is a row with a unique identifier (column “cid”), followed by 43,878 columns of genes expression values and 17 columns of metadata (columns “user_id”, “study_id”, “cell_id”, “organ”, “region”, “subregion”, “seq_tech”, “sample_status”, “donor_id”, “donor_gender”, “donor_age”, “original_name”, “cl_name”, “uhaf_name”, “tissue_type”, “cell_type”, and “marker_gene”) describing dataset-level information and cell-level information.

#### ECAUGT: the data access interface of uGT

Based on uGT, we developed a command-line tool “ECAUGT” (pronounced as “e-caught”) to query data from hECA for advanced users to implement *in data* cell sorting. Users can query the cells with the provided query conditions and download the selected data of these cells. For example, the combinatorial query of “all T cell subtypes located in the heart with PTPRC positive and CD3D or CD3E positive” can be written as the following logic expression:

(organ==Heart) && (cell_type == T cell) && (PTPRC > 0.5) && ((CD3D >= 0.5) || (CD3E >= 0.5))

hECA will return all cells that satisfy these conditions in a single downloadable file to users for further analysis. Information about the particular studies of the cells will also be provided to the users. Table S2 provides the syntax of the logic expressions in ECAUGT.

Function “query_cells()” will query cells with conditions on the columns of metadata and provide a user-friendly interface, with which users can combine multiple conditions into a logic expression in a structured string with logical operators ‘&&’ (for logical operation AND), ‘||’ (for logical operation OR), and ‘!’ (for logical operation NOT). Then “query_cells()” will return the cid list of the queried cells. Function “get_columnsbycell ()” will allow users to download data with this id list. Users can select columns of interest and add gene conditions in this function with the similar interface by “query_cells()”. The “get_columnsbycell ()” can provide downloaded data in two forms: a python list, where each element represents a cell, or a pandas.DataFrame object. Users can choose the form they want with the parameter “do_transform”. We also provide the parallel acceleration version with similar interface by “get_columnsbycell_para()”. Function “get_all_rows()” will provide the cid list of all cells in uGT and can be convenient when users require information of the whole hECA. Function “get_column_set()” receives a cid list and will provide all unique values in the selected column of these cells.

For users without much programming background, we provided a lightweight command-line tool “Cell_Download” to download data from hECA. Users first query cells in the website interface of hECA and download a cell id list file. Then “Cell_Download” only needs one-line command to assign the input and output path and will automatically download all columns of the selected cells in the id list and save the result in four files: a .csv file “metadata.csv” for columns of metadata, a .npz file for sparse expression matrix, and two .csv files for the row names and column names of this matrix. “ECAUGT” is available at PyPI (https://pypi.org/project/ECAUGT). Complete documentation of ECAUGT could be found at http://eca.xglab.tech/ecaugt/index.html.

#### The unified hierarchical cell annotation framework (uHAF)

To assemble cells into an atlas so that cell annotations from different studies can be aligned, we designed the index and coordinate system uHAF. It is a structured framework we designed for the hierarchical indexing and annotation of organ origins and cell types in hECA. We unified the information of anatomical structures, source organs, and cell types into a hierarchical knowledge graph. Users can assign annotations at multiple granularities with uHAF, depending on the quality of the data to be labeled.

We defined two types of entities using a controlled vocabulary, and composed two subgraphs in uHAF. Entities in the “macroscopic subgraph” include system, organ, anatomical region, and subregion information (see “uHAF macroscopic ontology” in https://github.com/XuegongLab/hECA/tree/main/UHAF). Entities in the “microscopic subgraph” include annotations of cells on their histological types (epithelial tissue, connective tissue, muscle tissue, and nerve tissue) and cell types or subtypes determined by molecular features (see “uHAF microscopic ontology” in https://github.com/XuegongLab/hECA/tree/main/UHAF). We defined two types of edges in the uHAF graph, “part of” and “is a”, to represent the hierarchical relations among the entities, and an extra “connect to” type of edge to tag attributes of the entities. For example, there is a “part of” edge from the entity “left ventricle” to the entity “heart”, and there is an “is a” edge from the entity “inhibitory neuron” to the entity “neuron”. If a cell type was present in certain organs, there are “part of” connections from cell type nodes to organ nodes, indicating the cell type composition of a macroscopic entity. For example, the entity “T cell” has a “part of” connection with the entity “left ventricle”, as well as “part of” connections to other anatomical units that have T cells in their tissues. We listed all the connections observed in our collected data of hECA v1.0 in the “uHAF macro-micro map” (https://github.com/XuegongLab/hECA/tree/main/UHAF).

The entities in the macroscopic and the microscopic subgraph are organized in a hierarchical directed acyclic graph (DAG) structure by manually surveying the canonical human anatomy structure and cell type names from classical medical textbooks, including *Junqueira*'*s Basic Histology: Text & Atlas* (Mescher, 2016), *Histology and Embryology* (in Chinese) (Tang and Zhang, 2013), *Systematic Anatomy* (in Chinese) (Bai and Ying, 2015), *Histology and Embryology* (in Chinese) (Li and Zeng, 2018) as well as several public studies and databases (Franzén et al., 2019), followed by confirmation and refinement from medical experts. We then organized the macroscopic and the microscopic subgraphs into ontologies with the protégé tool (https://protege.stanford.edu/).

The microscopic entities are attached with attributes “marker reference” consisting of marker genes by the “connect to” edges (see “uHAF marker reference” in https://github.com/XuegongLab/hECA/tree/main/UHAF). We adopted a combinatory approach to construct the marker reference by incorporating knowledge-based marker genes and data-derived DEGs. For those canonical cell-type-specific markers such as SLC17A7 of excitatory neurons, PTPRC of immune cells, etc., we added them to the “marker reference” directly. We also collected those markers reported in articles, including the original studies deposited in hECA, as well as well-established organ databases. Additionally, the dataset-wised analysis produced amounts of cluster-specific DEGs. For cell types whose marker genes were not given in the original studies, we surveyed for markers from multiple sources, including Gene Ontology (http://geneontology.org/), PanglaoDB (https://panglaodb.se/) (Franzén et al., 2019), the Human Protein Atlas (https://www.proteinatlas.org/) (Uhlén et al., 2015), and CellMarker (http://biocc.hrbmu.edu.cn/CellMarker/) (Zhang et al., 2018) to replenish the marker references. In most cases, we only considered the top10 DE-Gs for each cluster in each dataset. Such processes were implemented iteratively to curate the final marker references. The references will be continuously updated along with the release of new versions of hECA.

We provided the uHAF-related files at https://github.com/XuegongLab/hECA.

#### Cell identity assignment

We assigned an identity label from uHAF to every single cell collected. Each cell in hECA is annotated with two entities of the uHAF, one macroscopic and one microscopic. The annotation can be of different levels in the two hierarchies, depending on the information provided by the original data and the specificity of the marker genes.

##### uHAF name assignment

For each Seurat cluster, we identified the cluster-specific differentially expressed genes (DEGs) by the FindAllMarkers function. We referred to the marker reference to determine the cell type labels, and used the top ranked DEGs to further annotate the subtypes. We first determined the most general labels among the four tissue types (epithelial tissue, connective tissue, muscle tissue, nerve tissue). We then chose the deepest child cell type on which markers can be used to support the cell type assignment in the uHAF. In this way, we annotate each cluster “organ-tissue_type-cell_type-markers”, indicating the macroscopic and microscopic levels of the cluster. For cells that cannot be annotated based on available information, we named them as “Unclassified”. This label produced from uHAF is called “uHAF_name”. Table S3 lists the entity combinations that have been used in annotating the existing data in the current version of hECA. Users can use the uHAF to annotate their query cells in the same way.

##### Mapping uHAF names to cell ontology terms

We downloaded the basic Cell Ontology (Bard et al., 2005; Diehl et al., 2016) terms from the CL website (Cell Ontology - Summary | NCBO BioPortal (http://bioontology.org)), retained “Preferred Label”, “Definitions” and “Parents” (Table), and used the “Preferred Label” for CL term assignment. We converted the “uHAF_name” to “cl_name” by a combined strategy: We preferably used the Cell Ontology terms with the exact matching of the whole string of “cell_type”. For the “cell_type” that did not appear in the Cell Ontology terms, we further searched their parent “cell_type” in our uHAF until the Cell Ontology term was matched completely. For the remaining “cell_type”s, we manually determined the most similar Cell Ontology terms by ontologyIndex R package (Greene et al., 2017). If no term was found after these steps, we labeled them “none” (see Table S3).

#### Generation of quantitative portraits

We designed a portraiture system as a systematic way to characterize the complete properties of biological entities of all levels in hECA. There are three major types of biological entities in hECA: organs (including sub-organs), cell types (including subtypes), and genes. A complete quantitative portrait of a biological entity is its holographic picture of the entity at anatomical, cellular, and molecular levels. However, the quality and quantity of the currently available data in hECA are far from constructing such full portraits. Therefore, the quantitative portraits in hECA v1.0 only illustrated the idea of the portraiture system using the available information. They reflect more about the characteristics of the collected data of and related to each entity, rather than about the biological truth of the entity.

##### Organ portraits

A portrait of an organ is composed of 3 major parts: the cell composition viewer, the cell embedding viewer, and the organ hierarchy viewer. The cell composition viewer shows the counts and fractions of cell types observed in one organ’s datasets. It is notable that statistics in the organ portraits only reflect the counts/fractions of the collected cells, not the true counts/percentages of cell types in an organ. The embedding viewer visualizes cells of an organ with a 2-dimensional scattergram (UMAP/PCA/DensMAP for users to choose). This viewer supports coloring embedded cells by their cell types, sequencing technologies, original studies, and any given gene’s expression level. The organ hierarchy viewer shows the position of the organ in the uHAF macroscopic annotation system.

##### Cell type portraits

The cell type portrait depicts cells belonging to the same cell types/subtypes across all organs, and is composed of 4 major parts: cell distribution, marker genes, 2D visualization, and taxonomy relationship with other cell types. The cell distribution part describes the relationship of this cell type with organs, with bar plots showing the organ origin of cells in numbers and proportions. The marker gene part provides a table with genes highly expressed in this cell type, which were defined by comparing gene expression level with all other cell types using Seurat v3.2. We filtered out genes with adjusted p-value larger than 0.05 or expressed in fewer than 25% of cells in this cell type, and showed top 50 genes with the highest log fold changes. In the 2D visualization part, we plotted an interactive scatter plot showing the distribution and landscape of cells in this cell type. Embedded cells can be colored with their organs, sequencing technologies, original studies, and any given gene’s expression level. Like organ portraits, we also showed the cell type’s hierarchical relationship with other uHAF cell types.

##### Gene portraits

The portrait of a gene is composed of 2 major parts: basic gene information and gene expression distribution. In the basic gene information part, for each gene, we collected the full name of the gene, the position where the gene is on the genome, commonly used aliases of the gene, and a description that introduces the basic function of the gene. The “known as marker of” section denote cell types that highly express this gene, which is calculated by comparing the expression level of the gene in a cell type with it in other cells. For the gene expression distribution part, we first performed data normalization of all cells in uGT using function NormalizeData in Seurat v3.2. For each gene, we present its distribution in an organ or in a cell type by drawing a ridge plot. The ridge plot is fitted by expression value of the gene in the organ or cell type, while zero-value are truncated before fitting. The ridge plot also provides the median expression level and non-zero percentage.

#### The hECA website

We provided two portals for users to access hECA. One is a computer programing portal for users to access the data and do *in data* cell experiments using the ECAUGT package. The portal is at https://pypi.org/project/ECAUGT/. It is powerful but requires users to be comfortable with some programming skills. The other portal is a website at http://eca.xglab.tech/ with a graphic user interface (GUI) that enables both browsing hECA at all levels and searching the data for *in data* cell experiments. ECAUGT can also be accessed from the website portal.

The interactive functions of the hECA website (http://eca.xglab.tech/) are divided into four parts: “Cell sorting”, “uHAF cells”, “uHAF organs” and “gene portraits”, plus a link to the “ECAUGT” portal. Users can browse these functions anonymously, but signing in is needed to get the full service.

“Cell sorting” is the graphical interface for *in data* cell sorting in hECA v1.0. It supports flexible multi-step cell selection with all kinds of filters regarding to cell features (gene, cell type in uHAF, organ in uHAF, and other metadata). Filters can be combined with basic logic operators (AND, OR, NOT) to form complex logic expressions. Users can have a quick view of the selected data with real-time statistical analysis and visualization of the organ origin and cell type composition, and can adjust the sorting criteria accordingly if necessary. For more in-depth analysis, we provide the organ-wise cell type composition and gene expressions across cell types or organs and “FACS-like” plots visualization of expression correlation between any two genes. Cell sorting processes can be saved to users’ collections for future reference. After users selected their interested cell groups, a cid list can be downloaded for further data query with ECAUGT. Examples of *in data* cell sorting and vignettes are provided on the home page of the hECA website.

The design of having a user-login system allows users to save their searching history for possible future reuse. We also provided two anonymous test user accounts (usernames “test” and “test2”) for the reviewers’ convenience, both with password “123456”.

Cell types and organs are organized in uHAF DAG in hECA. The “uHAF cells” entry provides an interactive tree visualization of the cell types’ hierarchical relationships, which is the microscopic subgraph of uHAF. The “uHAF organ” entry provides a view of the macroscopic subgraph of uHAF. Each cell type or organ is assigned a unique uHAF ID with a brief description. We provide portraits for cell types with data available in the current version. Users can click “view details” to check the cell type portraits, including information of original organs, marker genes, and embedding view of the cell types. The plots can be colored by the organs, expression level of the selected gene, sequencing platform, or the original study. The organ portraits provide information on cell type composition (as reflected by the current data), similar embedding views, anatomy relationships and position in the uHAF.

The “gene portraits” entry allows users to select any particular gene and visualize the distribution of the gene in all organs and cell types (as reflected by the currently available data). The basic information includes the distribution of non-zero expression values in the organs and cell types, and the proportion of non-zero values (%Expr). Users should keep in mind the fact that the current scRNA-seq data is quite noisy and suffers from dropout events when using the information. The gene portraits also provide basic information of the gene collected from public databases and links to the corresponding pages at Genecard, NCBI, Ensembl, and Wikigenes.

#### Detailed descriptions of case studies

##### Case study 1

1. Get data

Using "query_cells" function in the ECAUGT package, we first sorted all cells in uGT with the label “T cell” and associated names (such as “CD4 T cell”, “CD8 T cell”, “Activated T cell”, etc.) across all organs (Figure S1A). To include cells that might be annotated to other cell types, we further searched for cells with normalized expression values of PTPRC, CD3D, or CD3E greater than 0.5 across all organs (Figures S1B and S1C). Then we filtered the cells by the expression of a list of negative markers such as COL1A1, and CD79A (the complete list provided in Table S4). After this step, we get a scanpy (Wolf et al., 2018) h5ad file for downstream experiments.

To refine the sorting results, we split cells by the dataset origin, conducted clustering analysis on the cells within each origin (Figure S1A), retained reliable cells with high CD3(CD3D, CD3E, or CD3G) expression levels (Figures S1B and S1C), and obtained a series of candidate clusters in each organ (Figures 2A and S2A). After these refining steps, we built an agile cell atlas of T cells across 18 organs (lung, pancreas, blood, liver, muscle, thymus, jejunum, rectum, colon, kidney, gallbladder, stomach, thyroid, intestine, spleen, bone marrow, eye, and vessel).

2. Cell type assignment and metabolism analysis

To assign accurate cell type annotations to the cells in the T cell atlas, we performed hierarchical clustering using signature genes CD4, CD8A, and CD8B, and divided the cells into 6 subgroups of 3 major groups (Figure S2A). The three major groups are CD4^+^, CD8^+^, and double-negative (CD4^−^and CD8^−^) T cells (Figure S2B). For the CD4^+^ and CD8^+^ groups, we further annotated the cells as resident memory T cells, central memory T cells, effector memory T cells, naïve T cells, cytotoxic T cells, etc., according to the positive markers listed in Table S5. Figures 2B and 2C show the UMAP of the CD4^+^ and CD8^+^ T cells with the subtype annotations and with the organ origin of the cells, respectively. Figure 2D shows the gene expression signatures of the identified T cell subtypes. For the double-negative cluster, we marked them as “T cells” without further analysis as there might be cells false negatives in CD4 or CD8 expression due to possible dropout events in scRNA-seq data.

To obtain the metabolic activity scores, we combined the cells within each candidate cluster and used the averaged expression values for the pathway analysis in the CD4 and CD8 group separately. We downloaded KEGG metabolism pathways and evaluated these candidate clusters' activation levels using GSVA. The z-score scaled GSVA scores were visualized in heatmaps (Figures 2E and 2F).

##### Case study 2

This is the tutorial using our Python package ECAUGT to download and analyze the cells of gene CD19 expressing.

1. Get data

Set the parameters for connecting the server and use the function “Setup_Client” to connect the server by providing the parameters above. The output box will return the information on whether the server is connected. Create a filter variable by function at first. For example, the string parameter "CD19 > 0.1" means the filter will filter out the cells of which gene CD19 expresses over 0.1. A data frame will be returned, the rows refer to the ids of the cells, and the columns refer to the information of each cell, like gene expression and belonging organ. By the “CD19 > 0.1” filter we got 2,566 cells back. Also, the visual website can be used to do the filtration by gene expression. Open the hECA website and set filters to get CD19^+^ cells. This step takes about 5 min. Then click “Download Data” and a file containing the ids of selected cells is downloaded. The gene expression data and metadata can also be acquired by setting appropriate filters and restricting the rows and the columns. By setting parameter “rows_to_get” to the ids of cells in which CD19 expresses and setting parameter “cols_to_get” to None, we can get all the information of the 2,566 cells mentioned before. The first 43,878 columns are the gene expression, and the last 18 columns are the metadata.

2. Analyze CD19^+^ data with scanpy

This part uses scanpy (Wolf et al., 2018) to analyze the data you download above. Set the basic parameters of scanpy to output detailed information, print the versions of packages, and set the dpi of figures. The gene expression data and metadata are input to function “sc.AnnData()” to get an AnnData object. The repetitive rows and the columns containing the NA values are removed first. We set 10,000 as the number of reads per cell to do the normalization. The following step is to logarithmize the data. Then we extract the highly variable genes and apply it to substitute features of the AnnData object. The genes are scaled to unit variance and we reduce the dimensionality of the data by running principal component analysis (PCA). With the PCA representation, we compute the neighborhood graph of cells and embed the graph in two dimensions using UMAP. The UMAP figure can be showed by different colors of different genes, different organs, or other labels. After the operations above, we can create a cross table of rows being organs and columns being cell types. By using the python package Pandas, the information of different pairs of organs to cell types can be checked.

#### Supplemental experiments

##### More results of data quality validation experiments using hECA heart data

In the experiment using hECA heart data for label transfer, we did 6 sets of experiments with the same task using different batches of data, differently-processed data, or different label transfer models to compare the performance of hECA library-size based adjustment on label transfer tasks. Results are shown in Table S8 and Figure S8.

Experiment 1: train the SingleR model with two batches

We used all 160,775 cells in two batches as the reference to training a SingleR (Aran et al., 2019) model and annotated the two query datasets. When training the model, we used the normalized data matrix with all 43,878 genes as input and outperformed the model trained on the highly-variable genes. The predicting accuracies were 0.9543 and 0.9036 on the two datasets, respectively.

Experiment 2: train the SingleR model with one batch separately

We used two batches of reference data separately to train two SingleR models and then annotated the query datasets with the two models. We used the same input dimensions and parameters as Experiment 1 when training the SingleR model. The predicting accuracies of batch 1 were 0.9485 and 0.8794 on the two datasets, respectively. The predicting accuracies of batch 2 were 0.9276 and 0.9198 on the two datasets, respectively. The accuracies on the individual batch references were lower than those on the assembled data for the dataset of (Litviňuková et al., 2020). The accuracy of batch 2 reference was slightly higher for the dataset of (Tucker et al., 2020).

Experiment 3: train SingleR model with Harmony corrected batches

We integrated the two batches in the reference data on the embedding space with Harmony (Korsunsky et al., 2019) and obtained the corrected data with PCA inverse transformation. We trained a SingleR model and annotated the two query datasets. The predicting accuracies were 0.9507 and 0.9076 on the two datasets, respectively. The performances were similar to Experiment 1, proving that the hECA preprocessing was as reliable as Harmony.

Experiment 4: train the KNN model with Harmony corrected batches

We merged the query data (one by one) with all reference data and integrated them in the embedding space with Harmony (Korsunsky et al., 2019). The same parameters were used as in Experiment 3. We conducted the KNN algorithm with BiocNeighbors (https://bioconductor.org/packages/release/bioc/html/BiocNeighbors.html) in the 100-dimension corrected-embedding space to find the nearest 10 cells in the reference data for each cell in the query data. These 10 neighbors vote for the final annotation of this cell. The predicting accuracies were 0.8642 and 0.8949 on the two datasets, respectively, lower than the above results.

Experiment 5: train SingleR model with scanorama corrected batches

We employed scanorama (Hie et al., 2018) as the batch-correction method to build reference using two batches. We scaled the gene expression values within each dataset and performed scanorama to obtain the batch-corrected values. For query datasets, we also used the scaled expression values. The predicting accuracies were 0.738 and 0.7283 on the two datasets, respectively, lower than the above results using hECA data.

Experiment 6: train the Seurat model with Seurat corrected batches

We conducted the anchor-based integration with Seurat on the reference data. We used the anchor-based label transfer interface in Seurat to transfer the label of the integrated reference data to the query data. The predicting accuracies were 0.9322 and 0.8949 on the two datasets, respectively, lower than the SingleR model but better than KNN.

Overall, the SingleR-based models reached similar performances and outperformed the other models. We observed that the SingleR model trained on the 2-batch reference data integrated in hECA reached higher accuracy and Kappa score than the two models trained on the 1-batch reference data. We also observed that the model trained on the harmony-corrected data showed similar performances despite the additional computation. The reversed-transformed data also lost their biological meanings. We observed a lower accuracy for the model trained on the scanorama-corrected data, showing less successful integration. These results confirmed the reliability and usability of the assembled data in hECA v1.0 and the effectiveness of the sequencing-depth-based correction for basic batch effects.

##### Results of data quality validation experiments on COVID-19 data

1. Data and study design

We performed label transfer experiments using 4 types of reference designs to show that the hECA data assembled from multiple batches can generate good transfer results. The experiments constructed 4 references using combinations of three batches of hECA assembled data: batch1 (GEO: GSE134355), batch2 (NCBI BioProject: PRJEB31843), batch3 (healthy control of GEO: GSE130148). Reference #1 contains batch1 only, Reference #2 contains batch2 only, and Reference #3 contains hECA normalized data of the three batches (1, 2, and 3). Reference #4 contains batch-integrated data of the three batches (1, 2, and 3). The single-cell transcriptomes in References #1, #2, and #3 were library-size normalized hECA assembled data, but the data in Reference #4 were batch-corrected by the Seurat integration algorithm. The labels in the hECA references were B cell, CD8 T cell, Dendritic cell, Macrophage, Mast cell, Megakaryocyte, Monocyte, Myeloid cell, NK cell, Neutrophilic granulocyte, Plasma B cell, and T cell.

2. Label transfer results

We evaluated the label transfer quality by calculating the classification accuracy and the adjusted Rand index (ARI). As References #1 and #2 contain data from single sources, their experiment results can be viewed as no-batch baselines. The accuracy was 82.84% in #1 and 92.53% in #2, and the ARI was 0.31 in #1 and 0.41 in #2. The performance in #1 was weaker because Microwell-seq generated data sparsity in #1 was higher. Reference #3 contains three batches of hECA data. Reference #4 has the same data as #3 but adjusted the expression values across the batches using the Seurat v3 CCA integration algorithm. Figure S12D visualized the label transfer results using Reference #3. Figures S12E–S12H visualized the fractions of the hECA labels transferred to the COVID-19 dataset under four designs, indicating the overall label transfer quality is good except for some confusing cell types (e.g., gdT and NKT, myeloid populations). Experiments using References #3 and #4 have similar accuracy performance compared with the baseline single-batch experiments. These observations confirmed that the library-size-based normalization methods used in hECA are sufficient to support label transferring when multiple batches exist.

3. DEG study design

We performed the hECA-control DEG analysis to study the use of hECA assembled data as an external healthy control group in disease studies. The DEG analysis identified the upregulated gene in the S (severe) group compared with the HC (healthy control) group in 4 cell types: macrophage, dendritic cell, CD8 T cell, and NK cell. The COVID-19 dataset contains original healthy control groups, we computed baseline DEGs for the 4 cell types using the COVID-19 HC groups. We designed four types of alternative healthy controls in the hECA data: cells from batch1 marked with "∗.1", cells from batch2 marked with "∗.2", cells from batches1 and 2 marked with "∗.1.2", and integrated cells from batches 1 and 2 marked with "∗.int" (Figure S12J). For instance, the "NK.1" group represents NK cells from batch1. "NK.1.2" group represents NK cells from batch1 and batch2, whose expression values are library-size normalized in hECA. "NK.int" represents NK cells from batch1 and batch2, whose expression values are corrected by the Seurat integration algorithm.

4. DEG results

We used the IOU values to check the consistency between hECA-control DEGs and the baseline DEGs. IOU values range from 0 to 1, which indicates "non-overlapping" to "identical," respectively. We obtained moderate IOU values (<0.6) because there were natural gaps between different data domains, and the order of fold-change values varied in different experiments. It is expected that hECA-control DEGs resemble the original baseline DEGs when the cell types are matched (diagonal positions in the heatmap). Among all comparisons, "Mac.2" and "Mac.covid" have the highest IOU value. The batch1 hECA-control (marked with "∗.1") generally have weaker consistencies because they are from different sequencing technology (Microwell-seq). The integration algorithm corrected batches (marked with "∗.int") shared fewer common genes with the baseline DEGs. We compared the gene set enrichment results of hECA-control DEGs and baseline DEGs in the macrophages. The enriched terms are similar in two settings, e.g., they both identified type II interferon signaling and Toll-like receptor signaling pathways. These observations illustrated that hECA data could serve as a useful control group in disease studies, and DEG testing should not be performed on data with extra batch correction.

#### Methods for data quality validation experiments

##### Using hECA heart data for label transfer and extended comparisons

1. Data preparation

For the reference data, we used part of the adult heart data in the hECA v1.0 containing 160,775 adult human heart cells in 2 batches (85,519 cells in the first batch and 75,256 cells in the second batch) from two donors and two sequencing technologies (10X V2 and 10X V3). For the query data, we selected the two heart cell atlas works not being collected in our hECA v1.0. For a better evaluation of the performance on the label transfer task, we unified the cell type annotations on the reference data and query data onto the heart cell types in our uHAF framework as ‘Adipocyte’, ‘Cardiomyocyte cell’, ‘Endothelial cell’, ‘Fibroblast’, ‘Macrophage’, ‘Neuron’, ‘Pericyte’, ‘Smooth muscle cell’ or ‘T cell’. We standardized the features onto 43,878 genes on the query data like we did in hECA to ensure the same gene numbers of the expression matrix for the label transfer models.

2. Training SingleR model

We used the default parameters of the ‘trainSingleR’ function in Experiments 1,2,3, and 5 to train SingleR models, where the model would calculate the DE genes with the Wilcox algorithm. Reference data for Experiments 1 and 2 were processed with the hECA standard pipeline.

3. Batch correction with Harmony

In Experiments 3 and 4, we took the first 100 PCs as the input of the Harmony algorithm. We set group.by.vars= "sample.source" where "sample.source" was the column name of the batches in the metadata, and this parameter controlled the variance to be removed during integration. We used theta = 5, lambda = 0.75 and max.iter.harmony = 20. These parameters controlled the proper intensity of data integration and were decided after some experiments. After the inversed transformation, we got the corrected scaled data. Then we multiplied the original standard deviation and added the original mean values to get the corrected and normalized data. We used the harmony-based-corrected data instead of our integrated data in hECA to train a SingleR model and annotate the query datasets.

4. Batch correction with KNN

In Experiment 5, when conducting KNN, we used the default parameters and the ‘MulticoreParam’ function for the BPPARAM parameter to accelerate the calculation.

5. Batch correction with Seurat

In Experiment 6, we used the top 50 PCs to integrate the reference data and the CCA algorithm in the ‘FindIntegrationAnchors’ function. When transferring the label from integrated reference data onto the query data, we used the top 30 PCs to find anchors and set the parameter n.trees in the ‘TransferData’ as 20.

##### Using hECA neuron data for label transfer

1. Data preparation

The reference was selected as all neuron and neuron subtypes in hECA v1.0, including “Neuron", "Excitatory neuron", "Granule cell", "Inhibitory neuron", "PV inhibitory neuron", "Purkinje cell", "5HT3aR expressing neuron", "VIP inhibitory neuron", "Sympathetic neuron", "Motor neuron", "Sensory neuron", "Bipolar cell”, “Amacrine cell", "Horizontal cell", "Ganglion cell", "Retinal ganglion cell", "Photoreceptor cell", "Rod cell", and "Cone cell", 185,419 cells in total. The query data were PsychEncode dataset (Wang et al., 2018) containing 27,412 cells from brain samples.

2. Training SingleR model

We performed basic Seurat analysis from quality control to cell clustering, and used well-known markers to manually assign query cells with uHAF cell type names for 22 clusters (Figure S9). These uHAF cell type names were regarded as true labels. Then we used SingleR (Aran et al., 2019) to annotate query cells with default parameters automatically.

##### Using hECA for label transfer and normal control in a COVID-19 study

1. Data preparation

To set the standard for comparison, we manually annotated cells in the COVID-19 study (GEO: GSE145926) in each sample with signature genes described in the original study (Figure S12C). We assigned the most granular label to a cell and labeled uncertain cells with a more general label. The cell-type labels included: B, Plasma, T, T CD4, T CD8, CD8 cytotoxic T, T cycling, Treg, NKT, NK, gdT (gamma-delta T), Neu (Neutrophil), macrophage, Mast, cDC1 (conventional dendritic cell type I), cDC2 (conventional dendritic cell type II), moDC (monocyte-derived dendritic cell), pDC (plasmacytoid dendritic cell), DC (dendritic cell), and Mye (myeloid cell).

2. Label transfer and Batch correction

In lung immune cell label transfer experiments (Figure S12), we used Seurat label transfer functions to classify the query COVID-19 study cells (GEO: GSE145926) using different references. The classification performances were evaluated by the accuracy (ACC) and adjusted Rand index (ARI). As the labels in the query and references are not the same, we made a table of correct transfers to compute the accuracy, which obeys biological "is-a" relationships. For example, it is allowed to predict a "cDC1" cell as a "Dendritic cell" because cDC1 is a kind of "Dendritic cell". We used the Seurat IntegrateData function with default parameters to produce the batch-corrected expression values across multiple data sources.

3. DEG analysis and enrichment analysis

In DEG analysis, the differentially expressed genes were identified using the FindMarkers function in the Seurat package and filtered with fold-change values > 2, adjusted p-value<0.05. For the IOU value evaluation, we took the intersection/union of two DEG lists and computed the quotients of their sizes. The gene set enrichment analysis was conducted using enrichR (Xie et al., 2021) with the aforementioned DEGs. The top 10 enriched terms in the WikiPathway 2021 Human database (Martens et al., 2020) with the least adjusted p-values were presented in the enrichment plots.

## Data Availability

This paper analyzes existing, publicly available data. These accession numbers for the original studies that generated the single-cell transcriptome datasets analyzed in this work can be found in Table S1. hECA database can be found in http://eca.xglab.tech/. All original code, including ECAUGT for database query and codes for case studies, has been deposited at https://pypi.org/project/ECAUGT/. Any additional information required to reanalyze the data reported in this paper is available from the lead contact upon request.

## Supplemental information

Document S1. Figures S1–S12

Table S9. Coverage of cell type-specific markers in neuron label transfer, related to STAR Methods

Table S1. Source datasets of hECA v1.0, related to Table 1

Table S2. Syntax of the logic expressions of ECAUGT in uGT, related to Figure 1

Table S3. uHAF annotation produces uHAF_name, related to Figure 1

Table S4. Details of case study 1: Negative markers for T cells, related to Figure 2

Table S5. Details of case study 1: Positive markers for T cells, related to Figure 2

Table S6. Details of case study 2: Organ and cell type table of CD19^+^ cells, related to Figure 3 The number of CD19 expressed cells from each cell type and each organ. Red and blue highlights were treatment effect and side effect, respectively.

Table S7. Details of case study 2: Organ and cell type table of CD22^+^ cells, related to Figure 3 The number of CD22 expressed cells from each cell type and each organ. Red and blue highlights were treatment effect and side effect, respectively.

Table S8. Data quality validation with heart label transfer task, related to STAR Methods The accuracy and kappa score of heart label transfer experiments with different integration methods and different label transfer models.
