# Supplementary material for: hECA: The cell-centric assembly of a cell atlas
Source: iScience. 2022 Apr 28;25(5):104318. doi: 10.1016/j.isci.2022.104318 (PMC9114628; doi:10.1016/j.isci.2022.104318)
Supplement: Document S1. Figures S1–S12 [file mmc1.pdf]

iScience, Volume *25*

Supplemental information

hECA: The cell-centric assembly of a cell atlas

Sijie Chen, Yanting Luo, Haoxiang Gao, Fanhong Li, Yixin Chen, Jiaqi Li, Renke You, Minsheng Hao, Haiyang Bian, Xi Xi, Wenrui Li, Weiyu Li, Mingli Ye, Qiuchen Meng, Ziheng Zou, Chen Li, Haochen Li, Yangyuan Zhang, Yanfei Cui, Lei Wei, Fufeng Chen, Xiaowo Wang, Hairong Lv, Kui Hua, Rui Jiang, and Xuegong Zhang

hECA: the cell-centric assembly of a cell atlas

Supplemental figures

- **Figure S1**. Filtering candidate T cell subpopulations, Related to Figure 2.
- **Figure S2**. CD4/CD8 T cell population definition, Related to Figure 2.
- **Figure S3**. Cell type and organ distribution of CD19 expressed cells, Related to Figure 3.
- **Figure S4**. Cell type and organ distribution of CD22 expressed cells, Related to Figure 3.
- **Figure S5**. Example gene portrait page of PTPRC, Related to Figure 1.
- **Figure S6**. Example cell type portrait page of Fibroblast, Related to Figure 1.
- **Figure S7**. Example organ portrait page of Brain, Related to Figure 1.
- **Figure S8**. Performance of hECA and other batch integration methods on heart label transfer task, Related to STAR Methods.
- **Figure S9**. Using hECA neuron data for label transfer, Related to STAR Methods.
- **Figure S10**. Overview of all cell types from different sample origins, Related to STAR Methods.
- **Figure S11**. Identification of 3 cell states in epithelial cells can also be retrieved by comparing with hECA data, Related to STAR Methods.
- **Figure S12**. Using hECA lung immune cells for label transfer and DEG finding, Related to STAR Methods.

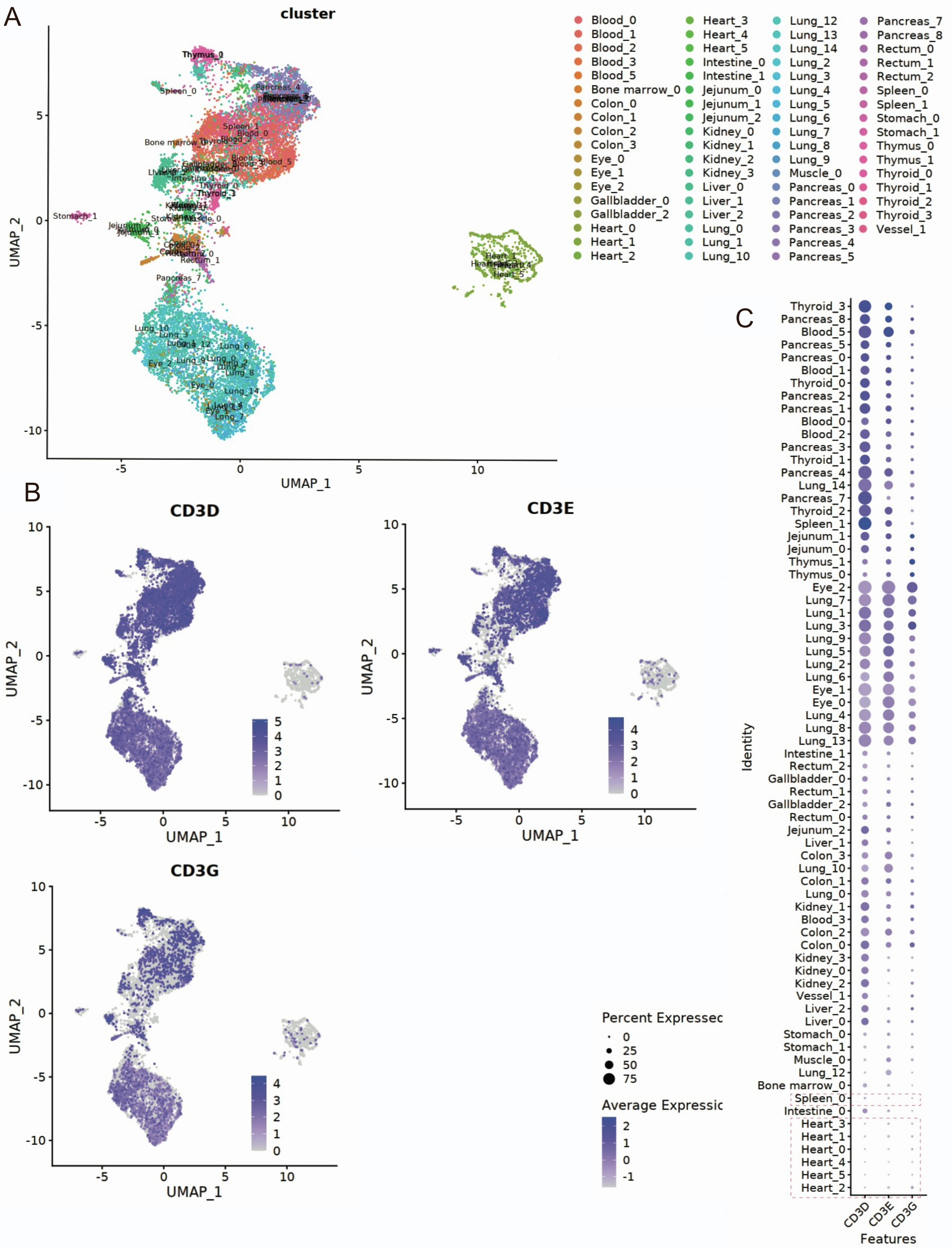

Figure S1. Filtering candidate T cell subpopulations, Related to Figure 2. (A) A UMAP showing the within-organ clustering results. (B) The general T cell markers' expressions (CD3D, CD3E, CD3G). (C) Per-cluster marker gene expressions.

A

B

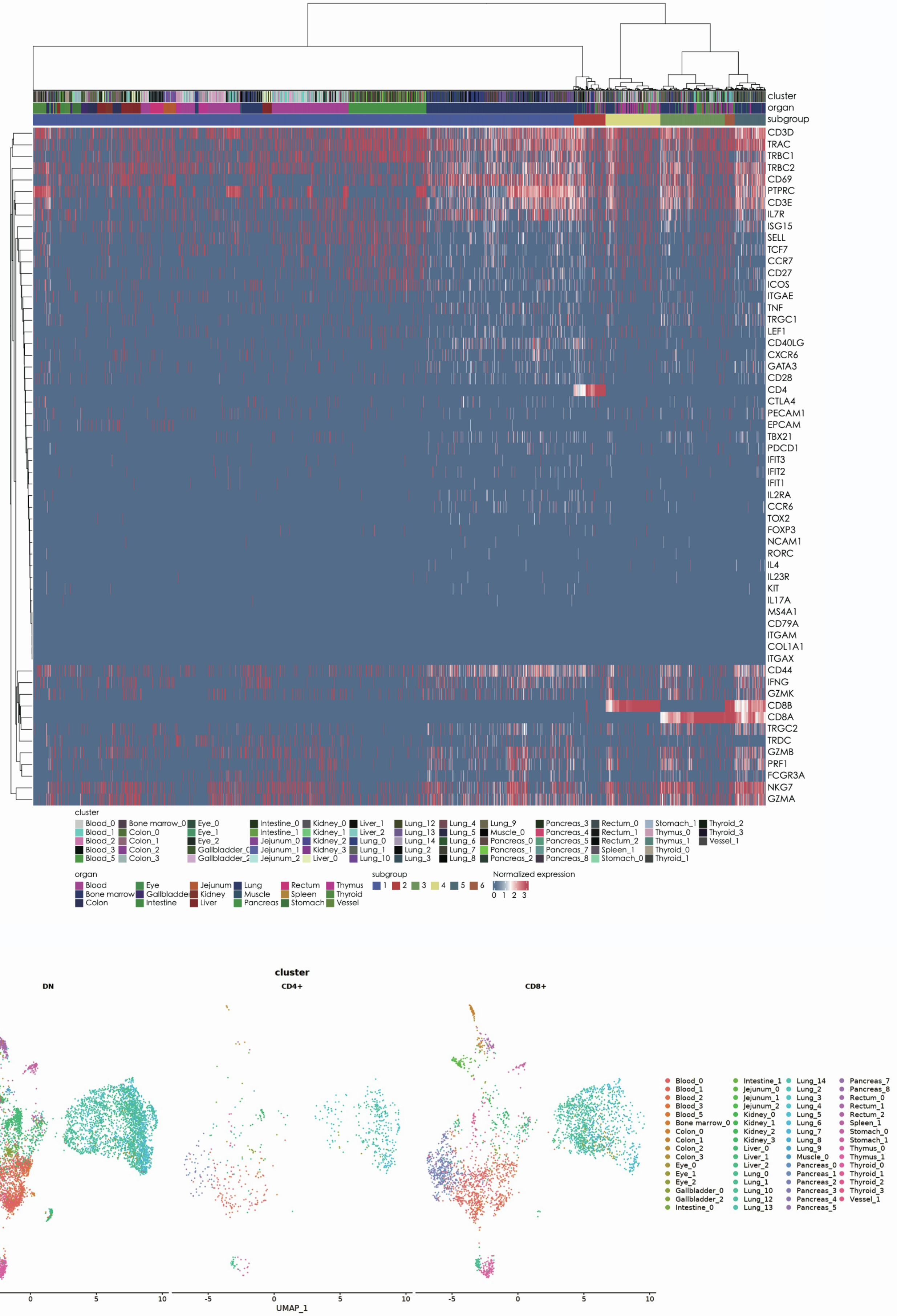

Figure S2. CD4/CD8 T cell population definition, Related to Figure 2. (A) A heatmap showing the hierarchical clustering results based on the CD4/CD8A/CD8B genes. Other T cell signature genes are also listed on the heatmap. (B) Split view of CD4 positive T cells, CD8 positive T cells, and double-negative T cells (Note that sequencing dropouts caused some falsely recognized double-negative T cells).

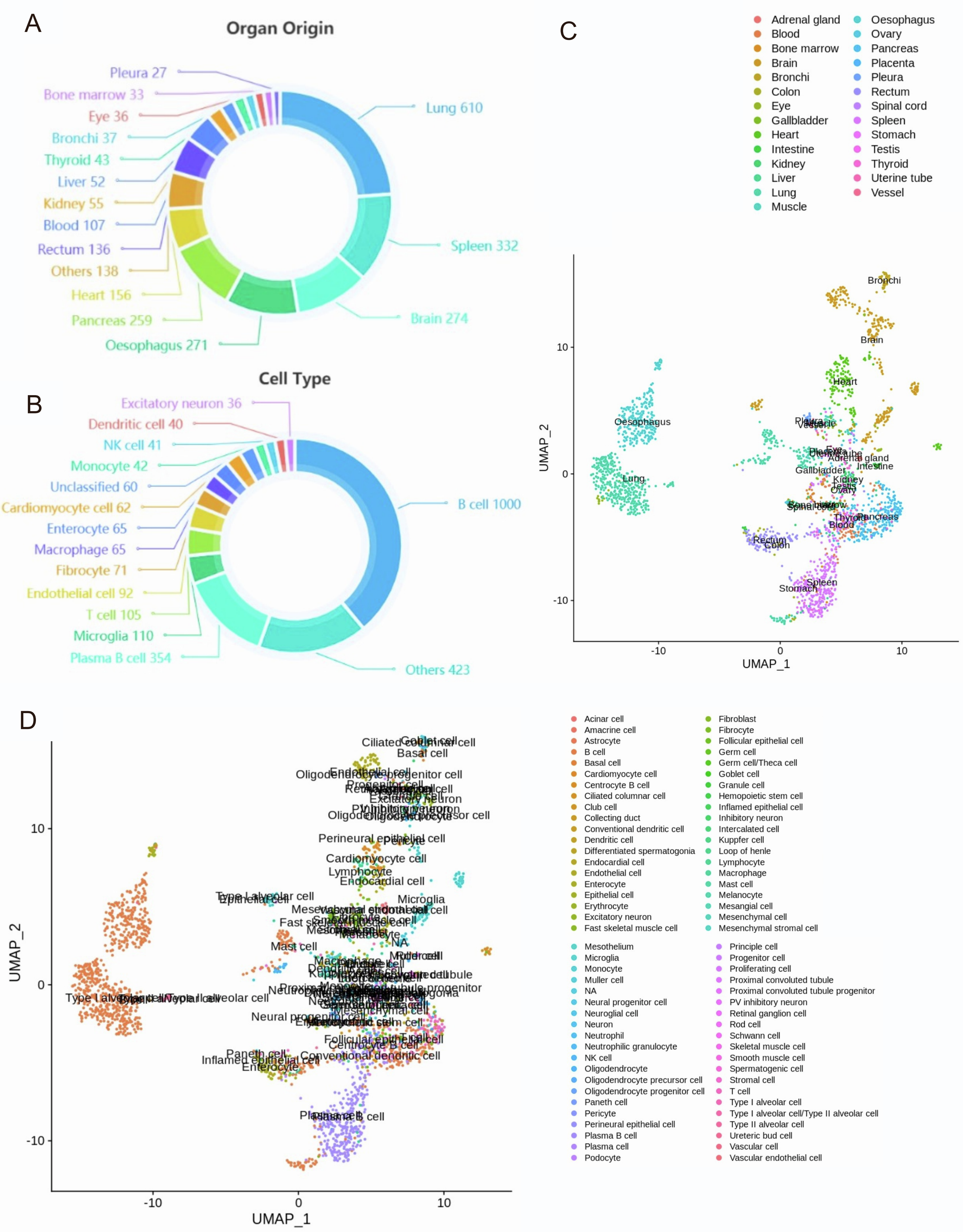

Figure S3. Cell type and organ distribution of CD19 expressed cells, Related to Figure 3. The number of CD19 expressed cells from different organs (A) and cell types (B). Visualization of CD19 expressed cells in UMAP labeling organs (C) and cell types (D).

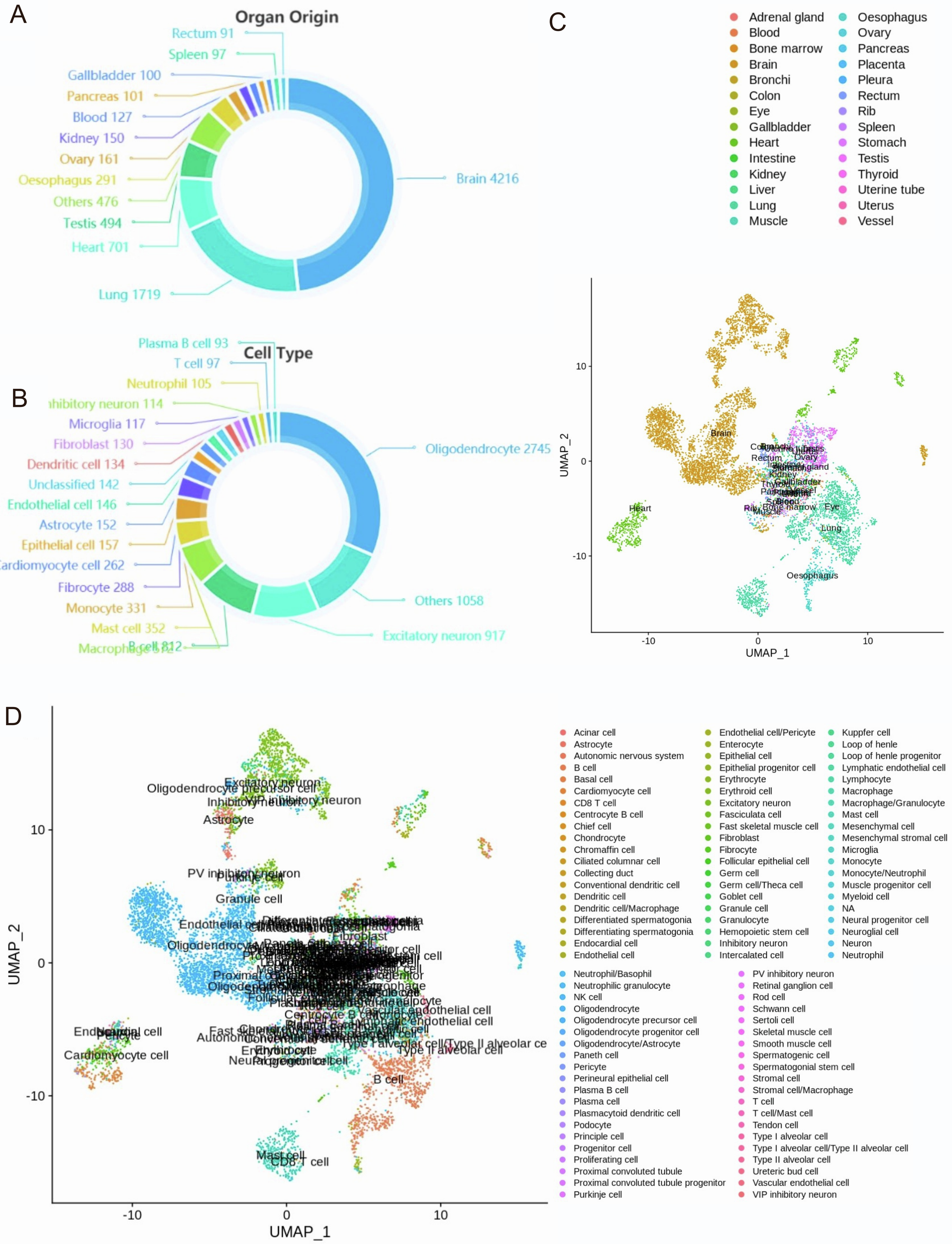

Figure S4. Cell type and organ distribution of CD22 expressed cells, Related to Figure 3. The number of CD22 expressed cells from different organs (A) and cell types (B). Visualization of CD22 expressed cells in UMAP labeling organs (C) and cell types (D).

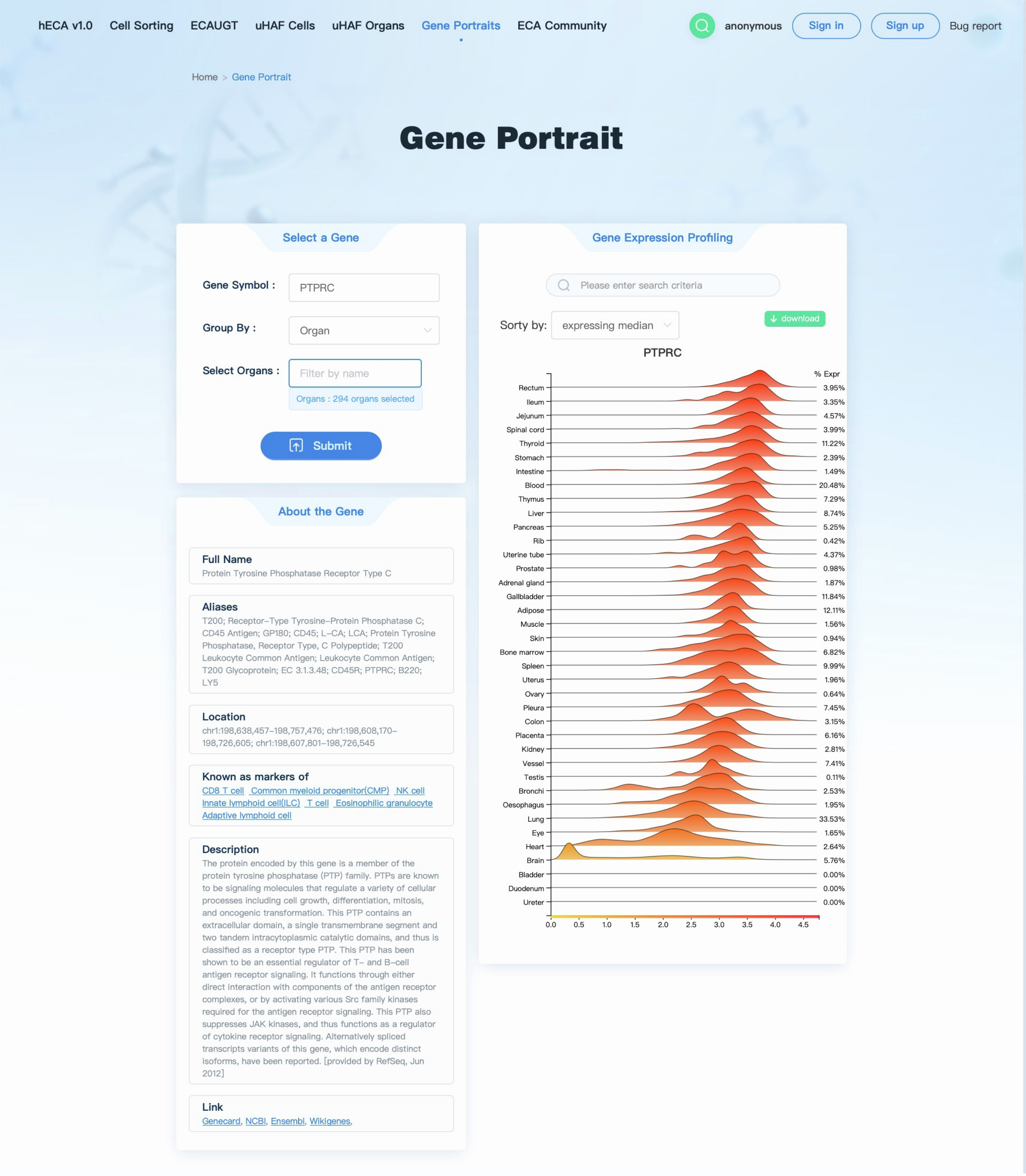

Figure S5. Example gene portrait page of PTPRC, Related to Figure 1. Basic information about PTPRC and distribution of expression level grouped by organs are shown in the “About the Gene” and “Gene Expression Profiling” panel, respectively. Users can select which gene to visualize and group by either organ or cell types in the “Select a Gene” panel.

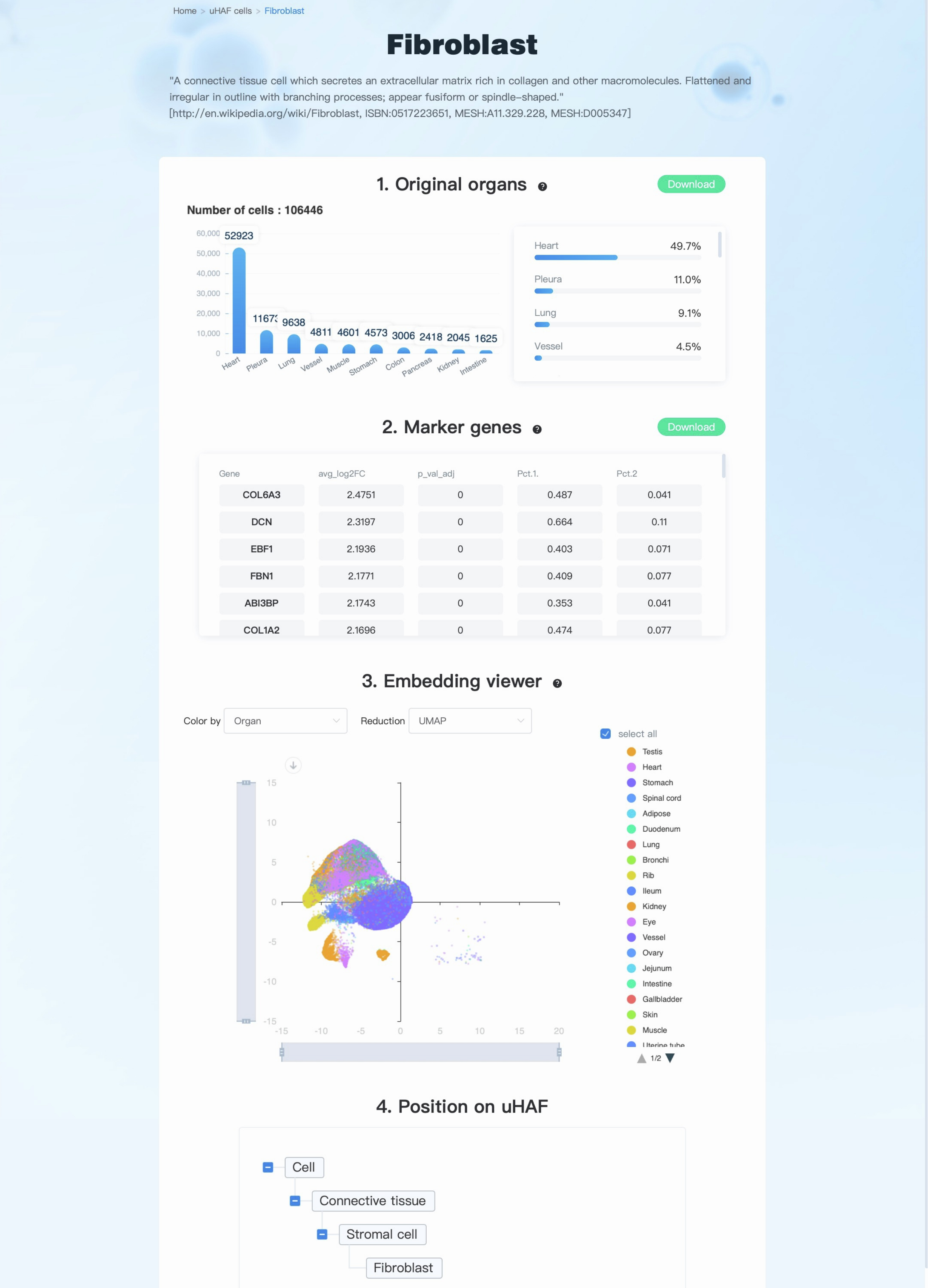

Figure S6. Example cell type portrait page of Fibroblast, Related to Figure 1. A brief description of Fibroblast is provided under the title. “Original organs” section shows the number and proportion of fibroblast cells in each organ. “Marker genes” section lists the differentially expressed genes in fibroblast and their characteristic expression ranges. “Embedding viewer” section visualizes the distribution of cells on 2D DensMAP space, colored by organs. It also provides 2D PCA and UMAP visualization of cells colored by sequencing technology, data source or expression level of certain gene. “Position on uHAF" marks the position of Fibroblast on the unified hierarchical annotation framework (uHAF).

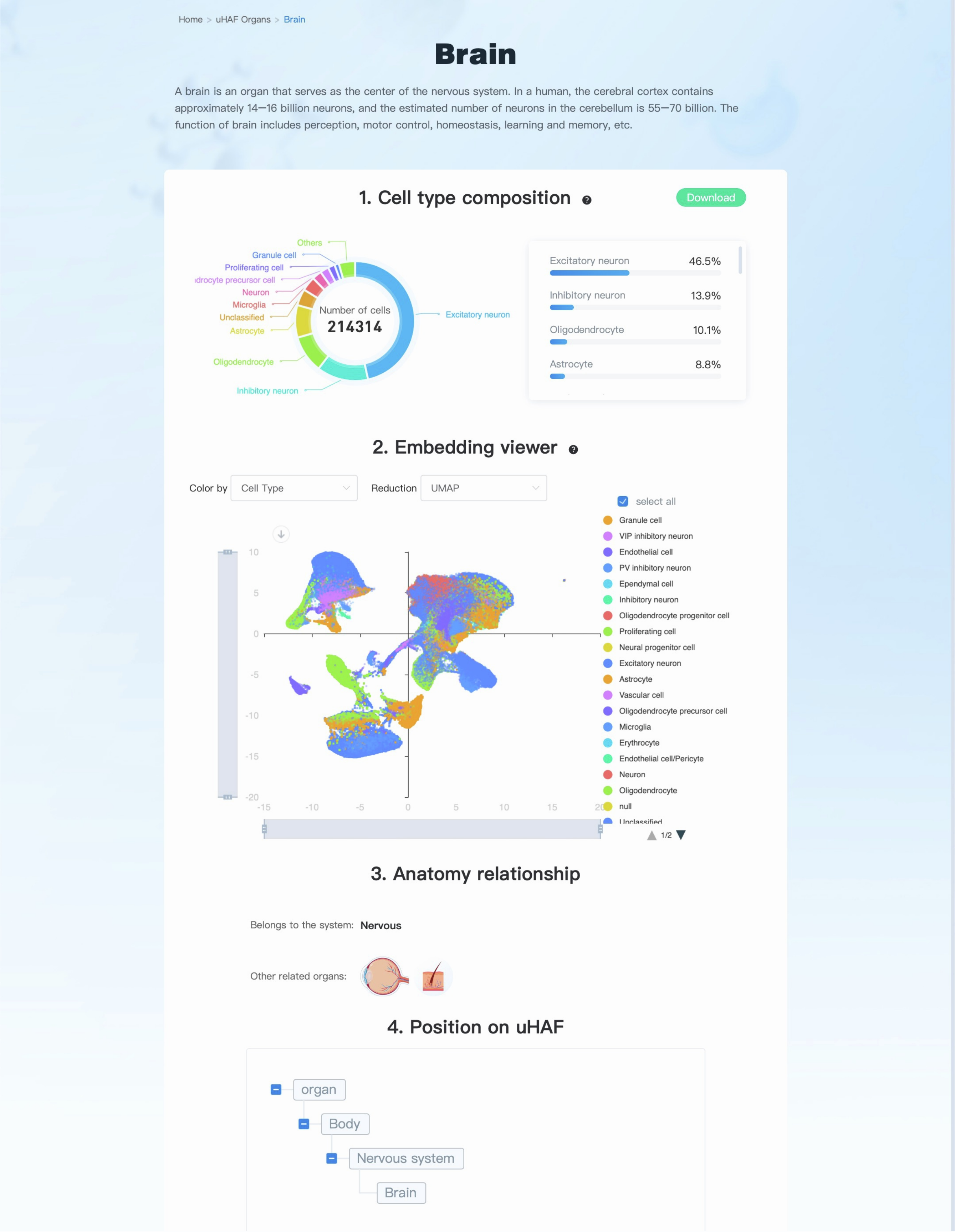

Figure S7. Example organ portrait page of Brain, Related to Figure 1. A brief description of Brain is provided under the title. “Cell type composition” section shows the number and proportion of brain cells in each cell type. “Embedding viewer” section visualizes the distribution of cells on 2D UMAP space, colored by cell types. It also provides 2D PCA and DensMAP visualization of cells colored by sequencing technology, data source or expression level of certain gene. “Anatomy relationship” shows the brain belongs to the nervous system and is related to eye and skin. “Position on uHAF" marks the position of Brain on the unified hierarchical annotation framework (uHAF).

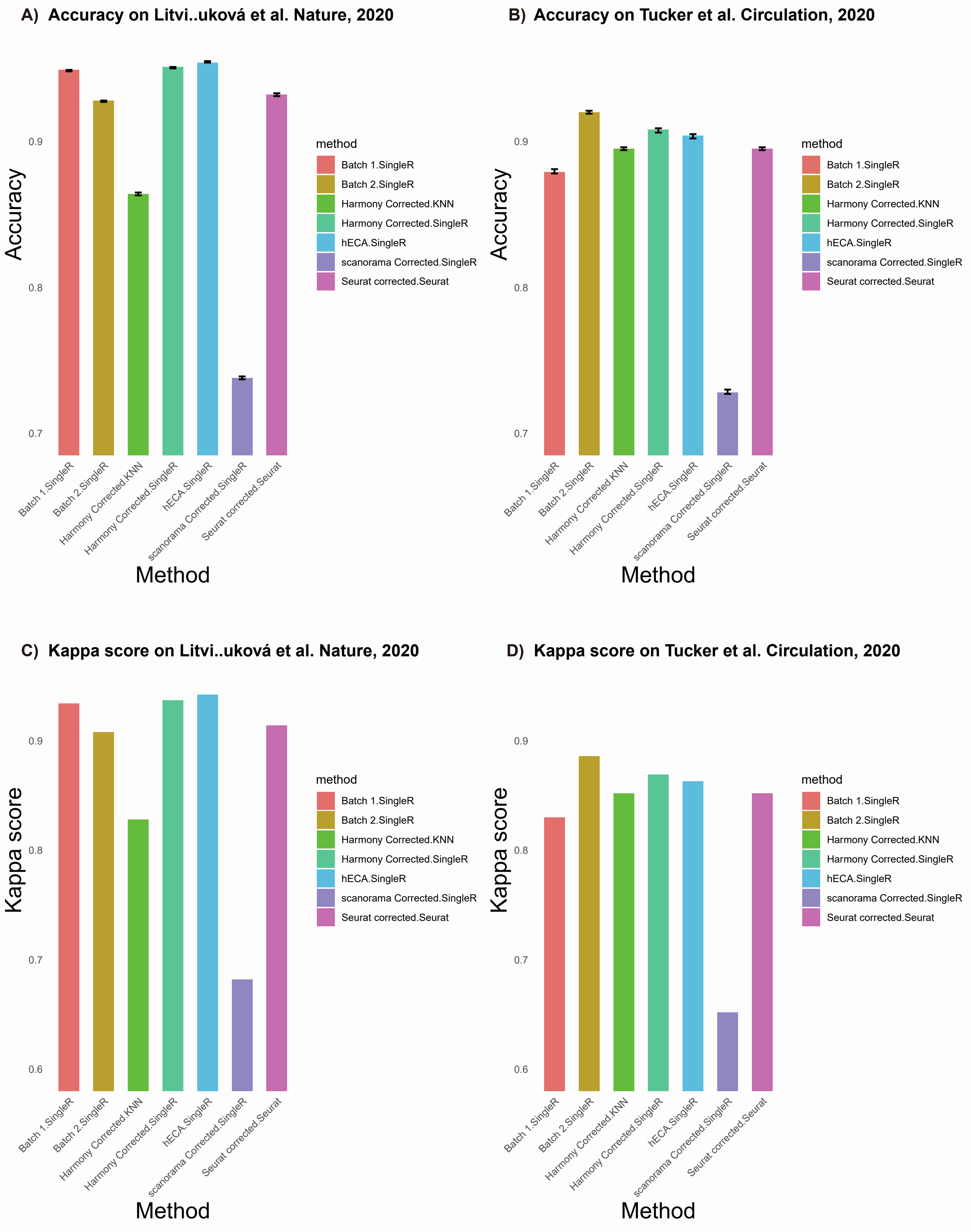

Figure S8. Performance of hECA and other batch integration methods on heart label transfer task, Related to STAR Methods. (A) The accuracy of 6 experiment on (Litviňuková et al., 2020) (B) The accuracy of 6 experiment on (Tucker et al., 2020). (C) The kappa score of 6 experiment on (Litviňuková et al., 2020) (D) The kappa score of 6 experiment on (Tucker et al., 2020).

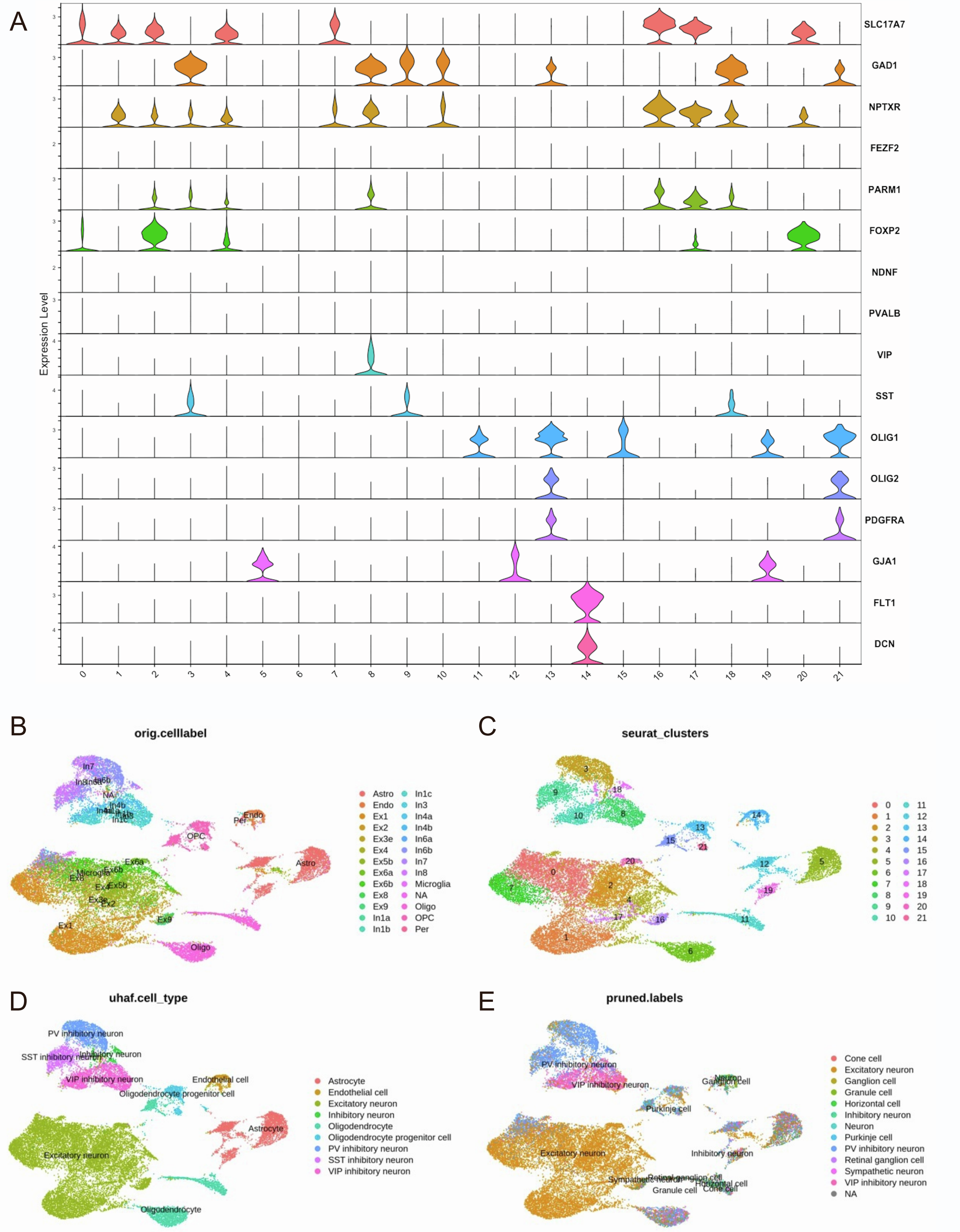

Figure S9. Using hECA neuron data for label transfer. (A) Well-known cell type markers used to determine the cell type , Related to STAR Methods. UMAP of query cells with the original cell type in uHAF name (B), Seurat cluster ID (C), uHAF cell type by manually annotation (D), and transferred labels predicted by SingleR (E).

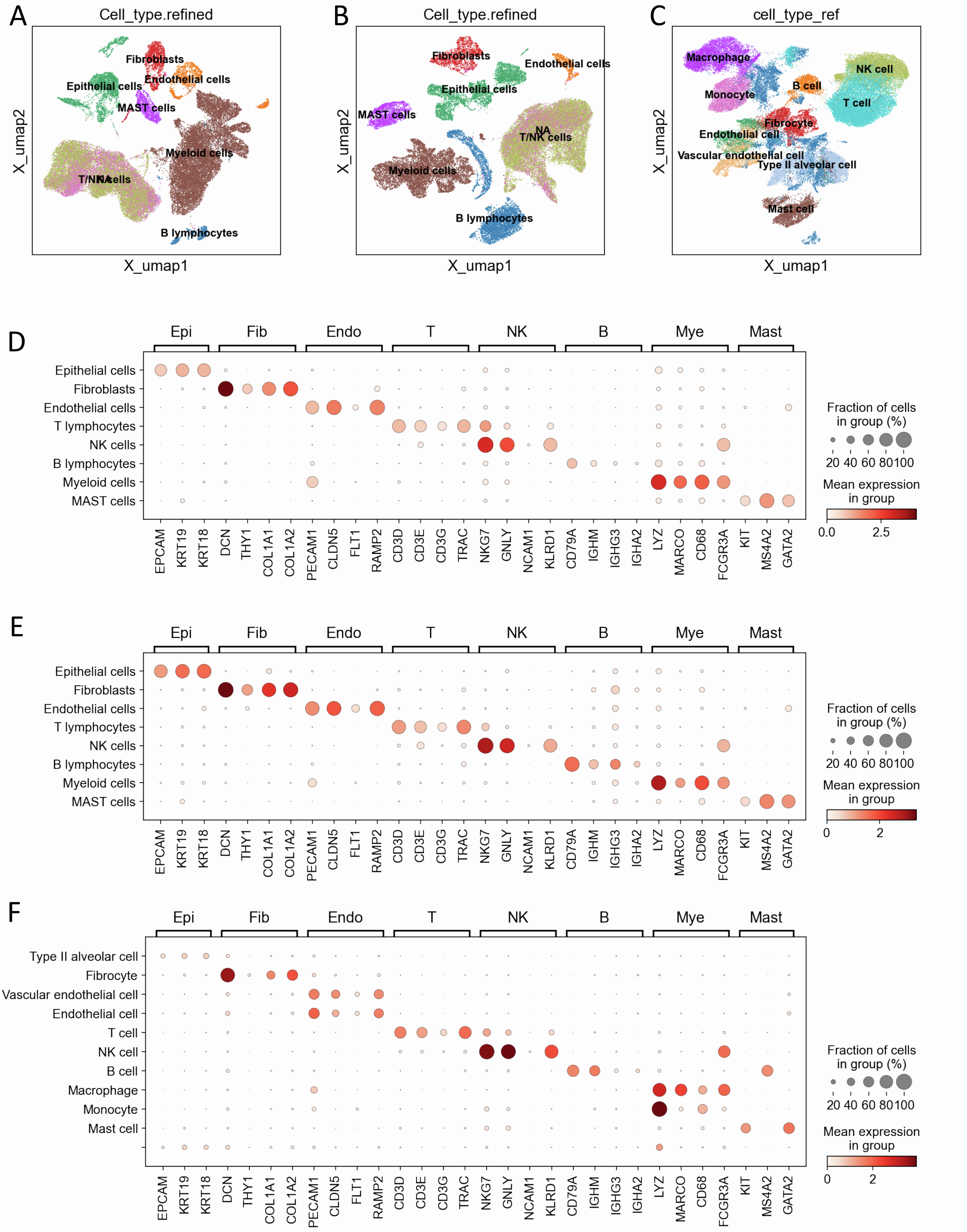

Figure S10. Overview of all cell types from different sample origins , Related to STAR Methods. (A-C) UMAP projection for normal lung (nLung), tumor lung (tLung) from paper and normal lung from hECA database (hECA). (D-F) Dotplot shows marker genes for major cell types in nLung, tLung and hECA.

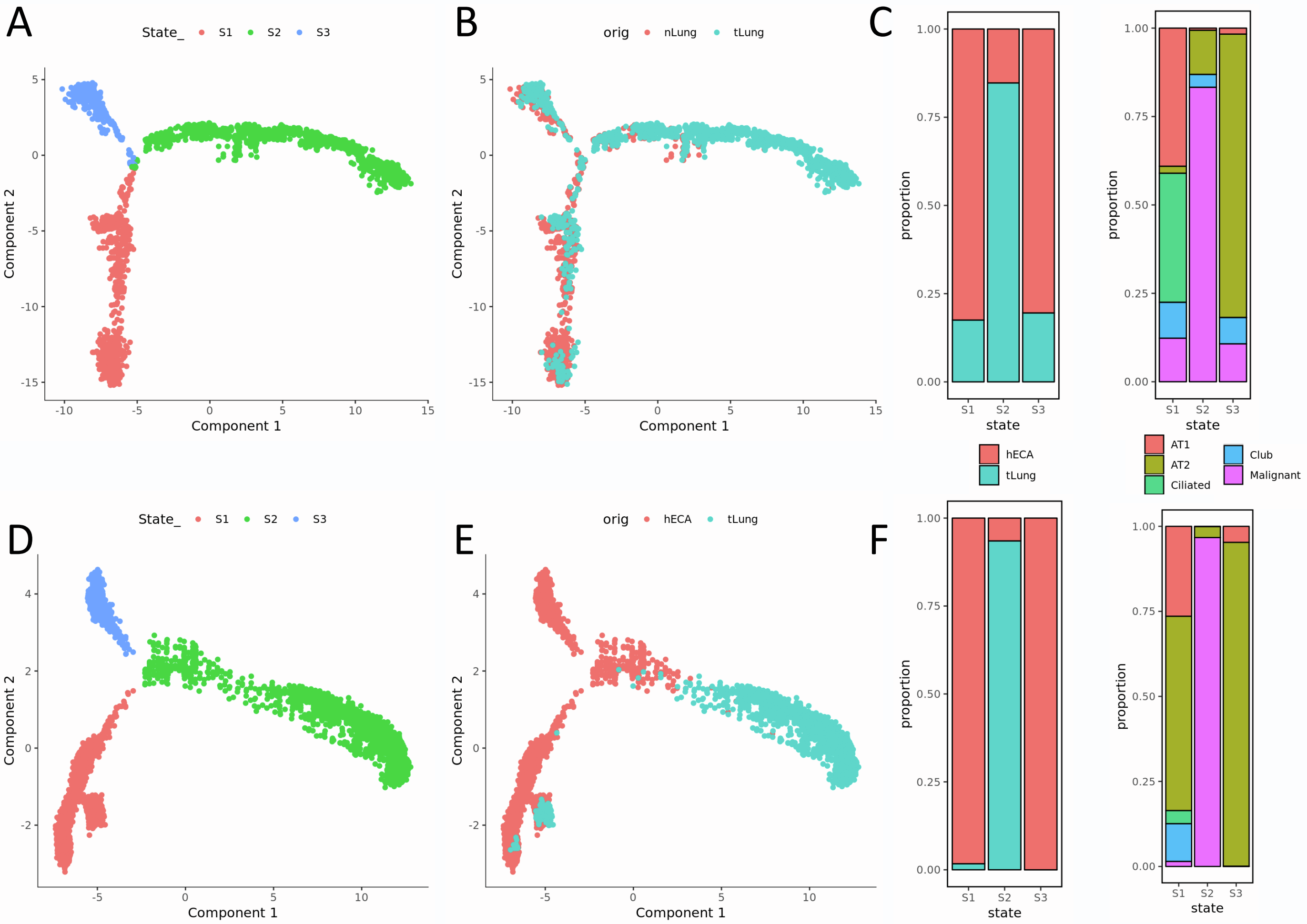

Figure S11. Identification of 3 cell states in epithelial cells can also be retrieved by comparing with hECA data, Related to STAR Methods. (A-B) monocle trajectory plot of epithelial cells from nLung and tLung, colored by cell states (A) and cell origins (B). (C) composition of cell origins for three cell states in A and B. (D-E) monocle trajectory plot of epithelial cells from hECA and tLung, colored by cell states (D) and sample origin (E). d)composition of cell origins for three cell states in D and E.

(A)

#	Reference Data	Methods	ACC	ARI
1	hECA batch1: Microwell-seq lung immune	Seurat v3 Label Transfer	82.84%	0.31
2	hECA batch2: 10xGenomics lung immune	Seurat v3 Label Transfer	92.53%	0.41
3	hECA unintegrated: lung immune	Seurat v3 Label Transfer	92.05%	0.37
4	hECA integrated: lung immune	Seurat v3 CCA integration + Label Transfer	92.50%	0.38

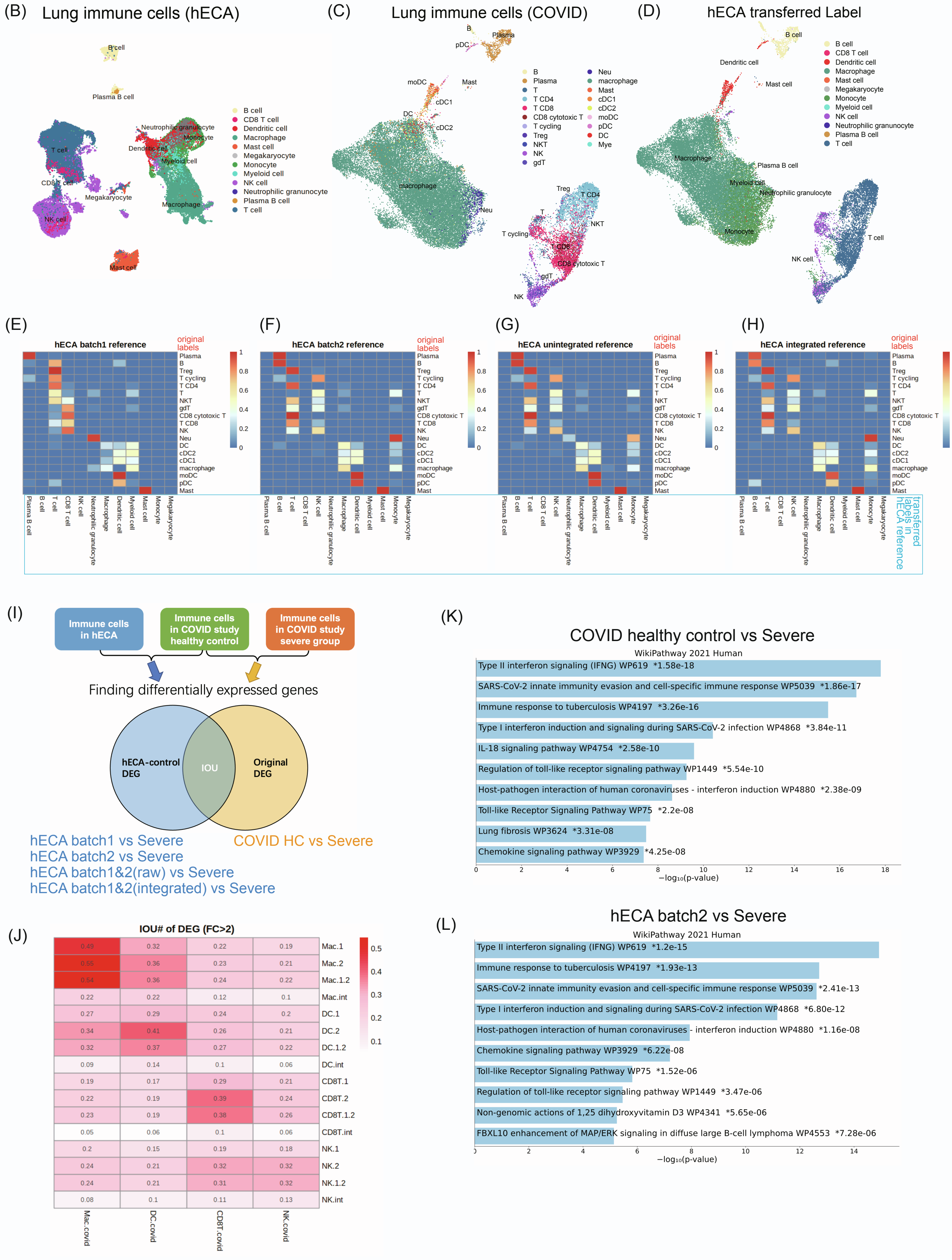

Figure S12. Using hECA lung immune cells for label transfer and DEG finding, Related to STAR Methods. (A) Label transfer experiment designs and performances. ACC: label transfer accuracy. ARI: adjusted Rand index (B) UMAP visualization of hECA lung immune cell-type labels. (C) UMAP visualization of a COVID-19 lung immune cell-type labels (GSE145926). COVID-19: coronavirus disease-2019 (D) UMAP visualization of cell-type labels transferred from hECA to the COVID-19 dataset. (E-H) Fractions of cells mapped to hECA labels in different cell types. The right row indices represent the labels derived from the original COVID-19 study. The bottom column indices represent the labels transferred from hECA. (I) Designs of the DEG (differentially expressed genes) finding experiments. We replaced the internal HC (healthy control) group in the COVID study with external hECA healthy control data and evaluated the consequences. (J) Consistency between the hECA-control DEGs and original DEGs. IOU: Intersection over union, the number of shared DEGs in two compared conditions divided by the number of total DEGs. (K-L) Pathway enrichment analysis of the original DEGs and the hECA-control DEGs.
